# German language questionnaires for assessing implementation constructs and outcomes of psychosocial and health-related interventions: a systematic review

**DOI:** 10.1186/s13012-018-0837-3

**Published:** 2018-12-12

**Authors:** Christina Kien, Marie-Therese Schultes, Monika Szelag, Rudolf Schoberberger, Gerald Gartlehner

**Affiliations:** 1 Department for Evidence-based Medicine and Clinical Epidemiology, Danube-University Krems, Dr.-Karl-Dorrek Strasse 30, 3500, Krems a.d. Donau, Austria; 2 Center for Public Health, Department of Social and Preventive Medicine, Medical University Vienna, Kinderspitalgasse 15, 1090 Wien, Austria; 3 Department of Applied Psychology: Work, Education, Economy, Faculty of Psychology, University of Vienna, Universitaetsstrasse 7, 1010 Vienna, Austria; 4 Department of Maternal and Child Health, Gillings School of Global Public Health, University of North Carolina at Chapel Hill, CB #7445 Rosenau, Chapel Hill, NC 27599-7445 USA; 5 RTI International-University of North Carolina at Chapel Hill Evidence-based Practice Center, Chapel Hill, 27599-7445 NC USA

**Keywords:** Implementation variables, Psychometric properties, Test-theoretical criteria, Instrument, Questionnaires

## Abstract

**Background:**

Over the past years, implementation science has gained more and more importance in German-speaking countries. Reliable and valid questionnaires are needed for evaluating the implementation of evidence-based practices. On an international level, several initiatives focused on the identification of questionnaires used in English-speaking countries but limited their search processes to mental health and public health settings. Our aim was to identify questionnaires used in German-speaking countries measuring the implementation of interventions in public health and health care settings in general and to assess their psychometric properties.

**Methods:**

We searched five different bibliographic databases (from 1985 to August 2017) and used several other search strategies (e.g., reference lists, forward citation) to obtain our data. We assessed the instruments, which were identified in an independent dual review process, using 12 psychometric rating criteria. Finally, we mapped the instruments’ scales and subscales in regard to the constructs of the Consolidated Framework for Implementation Research (CFIR) and the Implementation Outcome Framework (IOF).

**Results:**

We identified 31 unique instruments available for the assessment of implementation science constructs. Hospitals and other health care settings were the ones most often investigated (23 instruments), while education and childcare settings, workplace settings, and community settings lacked published instruments. *Internal consistency*, *face* and *content validity*, *usability*, and *structural validity* were the aspects most often described. However, most studies did not report on *test-retest reliability*, *known-groups validity*, *predictive criterion validity*, or *responsiveness*. Overall, the majority of studies did not reveal high-quality instruments, especially regarding the psychometric criteria *internal consistency*, *structural validity*, and *criterion validity*. In addition, we seldom detected instruments operationalizing the CFIR domains *intervention characteristics*, *outer setting*, and *process*, and the IOF constructs *adoption*, *fidelity*, *penetration*, and *sustainability.*

**Conclusions:**

Overall, a sustained and continuous effort is needed to improve the reliability and validity of existing instruments to new ones. Instruments applicable to the assessment of implementation constructs in public health and community settings are urgently needed.

**Trial registration:**

The systematic review protocol was registered in PROSPERO on October 19, 2017, under the following number: CRD42017075208.

**Electronic supplementary material:**

The online version of this article (10.1186/s13012-018-0837-3) contains supplementary material, which is available to authorized users.

## Background

Clinical and health services research often takes up to 17 years or even fails altogether to translate into policy and practice [1, 2] resulting in an ineffective use of resources. Furthermore, in German-speaking countries, as in the rest of the world, there is a need to assess the implementation of evidence-based practices (EBP). Only if we can assess whether interventions are implemented properly will we know if they produce genuine public health effects [3]. In recent years, implementation science has increasingly relied on the use of theories, frameworks, and models to guide the implementation of evidence-based programs and to improve the planning of evaluation studies [4–6]. To support this use, overviews of theories [4, 7–10] as well as criteria and guidelines on how to select theories [5] have been published.

Despite this orientation towards theories, reliable and valid questionnaires to draw conclusions from evaluation studies would allow for greater advancements in implementation science and assist in closing the evidence-practice gap [11]. Knowledge can only be advanced when comparable, reliable, and valid questionnaires (i.e., instruments) are used to study implementation constructs (i.e., abstract phenomena that are not directly observable) and strategies [12]. Recent systematic reviews contributed to the field’s development by revealing which theoretical domains and constructs associated with the adoption and implementation of programs could be assessed in a reliable and valid way [13–18]. Some limitations of previously conducted reviews [19] include the incomplete reporting of the instruments’ psychometric properties (e.g., test-theoretical parameters, such as reliability and validity) and having an exclusive focus on their use in hospital and health care settings [20].

More recently, one initiative [21] and one systematic review [19] provided a more comprehensive perspective on the instruments’ psychometric properties and covered a broad range of theoretical domains and constructs. The Society for Implementation Research Collaboration (SIRC) focused on the mental health care setting in their Instrument Review Project [21]. The review by Clinton-McHarg and colleagues [19] complemented this by concentrating on the public health care setting. Members of the SIRC Instrument Review Project team identified over 420 instruments [21] related to the Consolidated Framework for Implementation Research (CFIR) [22] and the review by Clinton-McHarg’s group identified around 50 instruments related to CFIR’s constructs. CFIR is considered to be a determinant framework. When developing CFIR, researchers analyzed the definitions and the terminology of several existing frameworks and theories and finally presented factors that act as barriers or enablers of the implementation process [4]. Overall, CFIR comprises 39 different constructs grouped into five different domains relating to intervention characteristics (e.g., evidence strength and quality, and complexity), outer setting (e.g., patient needs and resources), inner setting (e.g., implementation climate, network, and communication), characteristics of individuals (e.g., knowledge and beliefs about the intervention, self-efficacy), and process (e.g., planning, engaging). Furthermore, the SIRC Instrument Review Project team located more than 100 instruments [23] addressing domains of the Implementation Outcomes Framework (IOF) [24]. This framework covers eight different implementation outcomes. They are seen as revealing the effects of the implementation process and focus on the following aspects: acceptability, adoption, appropriateness, feasibility, fidelity, implementation cost, penetration, and sustainability of the intervention. Although both reviews applied comprehensive search strategies and assessment approaches, neither took into account the general hospital and health care settings besides mental health interventions [21], and Clinton-McHarg’s group [19] did not include the domains of IOF as relevant outcomes.

Since implementation science is becoming more prevalent in German-speaking countries [25, 26], a systematic search for instruments that can be used with German-speaking populations is highly relevant. Furthermore, as most tools available for judging the influence of contextual factors or the implementation process on the effect of interventions have been developed in English-speaking countries, it remains hitherto unclear as to how many questionnaires might be available for this purpose in German. Unfortunately, the aforementioned reviews located only a single instrument developed and used in German. Since it would be vital for oversight bodies in German-speaking countries to possess tools so as to judge implementation outcomes, there is an urgent need to determine the number and quality of available instruments for this purpose.

To the best of our knowledge, no previous review has been conducted focusing on implementation constructs assessing instruments that are available in German and designed for use in public health and health care settings. The aims of this review—following a similar approach to those already conducted in this field [19, 21, 23]—were firstly to identify quantitative instruments assessing constructs described in CFIR [22] and IOF [24], which have been applied within a German-speaking population, and secondly to survey the psychometric properties of the identified instruments. CFIR and IOF were chosen because of their comprehensiveness and high usage rate in the evaluation of interventions [5].

## Methods

We registered this review’s protocol in PROSPERO (International Prospective Register of Systematic Reviews) under the registration number CRD42017075208. The design of the systematic review follows SIRC’s Instrument Review Project [21] and Clinton-McHarg’s group approach [19].

### Eligibility criteria

We included publications if they (1) were published in peer-reviewed journals, (2) reported on quantitative instruments, such as questionnaires or surveys, which (3) were applied to assess the implementation of a specific psychosocial or health-related innovation or intervention, (4) assessed at least one of the 38 CFIR^1^ [22] or one of the eight IOF [24] constructs, and (5) were developed for the use in public health (e.g., child care or community centers, schools, universities, workplaces, and prisons) and health care settings (e.g., hospitals, general practice, allied health facilities such as physiotherapy or dental practices, rehabilitation centers, psychiatric facilities). Furthermore, these instruments should have at least one aspect of reliability or validity assessed and should have been completed by German-speaking facilitators or participants of the interventions. We included the following psychometric properties in our review: internal consistency, construct validity, criterion validity, structural validity, responsiveness, face and content validity, norms, usability, and test-retest reliability.

### Data sources and search process

We searched MEDLINE (via PubMed), PsycINFO (Ovid), PSYNDEX plus Literature and Audiovisual Media, PSYNDEXplus Tests and Education Resources Information Center (ERIC) from 1985 until August 2017. We assumed that no instrument would be published before 1985 as implementation science evolved later [21]. We selected these five databases, as they index relevant journals reporting on the evaluation of implementation of psychosocial or health-related interventions. The search strategy entailed the following elements and several variations of the search terms for a keyword search as well as for a search with Medical Subject Headings (MeSH) terms: (1) questionnaire, (2) psychometric properties, (3) intervention, and (4) implementation. We limited electronic searches to English and German as well as to human populations. Furthermore, we limited the search results to references with at least one author residing in a German-speaking country (“Affiliation” set to an institution in Austria, Germany, or Switzerland). We assumed that authors residing in German-speaking countries most likely would have tested their instruments on German-speaking population samples. We amended the search strategy developed in MEDLINE (via PubMed) to other databases. The detailed search strategy is presented in Additional file 1.

Additionally, we promoted our research project via a snowball sampling e-mail procedure to German-speaking experts in the field of implementation science and via an entry in the German-speaking Implementation Association’s [26] newsletter, intending to identify further relevant publications. We also used several recent systematic reviews on this topic [13, 15, 17, 19, 20, 23, 27] to check via forward citation tracking using Scopus if the instruments had been applied in German-speaking countries.

In a second step, we used already located instruments and continued the search process to detect further publications reporting on psychometric properties of these instruments. We searched the Scopus database by entering the name of the instrument in the search field and by using the forward citation tracking link of the source article.

### Study selection

Two investigators independently reviewed abstracts and full-text articles according to a priori defined eligibility criteria and solved conflicts by discussion. All reviewers piloted the abstract and full-text review forms to test the applicability of inclusion and exclusion criteria. This process led to the refinement of the definitions of psychosocial and health-related interventions. The abstract review was carried out in AbstrackR [28]. We managed and saved all results of the abstract and full-text review including information on the reasons for exclusion in the full-text review in an Endnote database.

### Data extraction and rating process

We piloted and improved the layout of the sheets and the rules for data extraction according to the feedback of the research team (e.g., how to deal with two studies reported in one paper). One reviewer extracted the pre-specified relevant data from eligible publications and a second reviewer checked the data for correctness. The reviewers solved discrepancies by consensus or by involving a third reviewer. We extracted data points relating to the development and assessment process of the instrument, to the description of the instrument, and to its psychometric characteristics.

#### Development and assessment process

This includes research setting, sample (gender and profession of participants answering the questionnaire), study characteristics (response rate), country where the instrument was developed, and characteristics of the intervention being assessed.

#### Description of the instrument

This embodies the name, abbreviation and aim of the instrument, number and names of subscales, and number of items.

#### Psychometric properties

This includes internal consistency (i.e., reliability), construct validity (convergent, discriminant, and known-groups), criterion validity (predictive and concurrent), structural validity (i.e., dimensionality), responsiveness, norms, and usability. Following Clinton-McHarg’s group approach [19], we also included information on test-retest reliability, face, and content validity. Lewis and colleagues described evidence-based assessment (EBA) rating criteria that have undergone a thorough development process [21, 29] and were compiled in the Psychometric and Pragmatic Evidence Rating Scale (PAPERS). The scale includes six different rating levels with clearly defined cut-off values ranging from “− 1—poor”, and “0—no information available” to “4—excellent” for psychometric properties (Additional file 2). Two different investigators independently rated the psychometric properties for each individual study. Instruments that were assessed in more than one study received an overall rating applying the worst score counts approach (i.e., the worst rating achieved in different studies represented the final vote). We deviated from this practice in our assessment of the domain “norms”. There, we used the best score counts approach as all interested researchers have access to the best available information.

After the assessment of the psychometric properties, one reviewer assigned the scale and subscales of the included instruments to 38 CFIR constructs and subscales [22] and eight IOF constructs [24]. A second reviewer checked this assignment. The mapping process focused on the description of the subscales and scales and not on the items.

### Analyses and reporting of the data

We reported on the number of identified instruments and further used descriptive statistics (i.e., frequencies, mean, median, standard deviation, and range) to inform about the psychometric properties of the instruments and the results of the mapping process (assigning scales to the CFIR and IOF constructs). We used Microsoft Excel 2010 for calculating the descriptive statistics.

## Results

First, we describe the results of the search process. Then, we present the identified instruments and their psychometric properties. Finally, we display the instruments’ mapping against CFIR and IOF constructs.

### Results of the search process

**Fig. 1:**
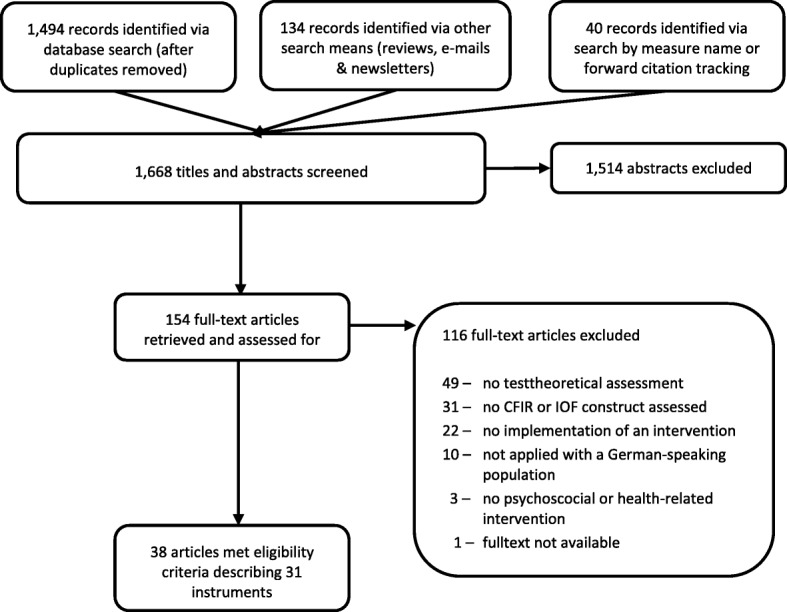
PRISMA flow diagram of the study selection process

Our database search yielded 38 articles [30–67] reporting on the psychometric properties of 31 different instruments. The detailed flow of the literature selection process is depicted in Fig. 1. The majority of the instruments (23/31; 74%) were developed for the use in hospital and health care settings [30–35, 38, 41–43, 47–51, 53–59, 63, 64, 67, 68]. Two instruments each were applied in the education [36, 52] and workplace settings [39, 45], and the psychometric properties of four instruments [37, 40, 44, 46, 55, 60–62, 65, 66] were assessed in more than one different setting (Table 1). Diverse interventions ranging from psychological and drug treatments to organization-wide implementation of quality improvement systems were evaluated using the identified instruments. Several questionnaires dealt with the assessment of web-based or technology-focused interventions. The number of subscales varied between one and 16 and the number of items per instrument ranged from two to 67 [37, 52]. The majority of the studies were conducted in Germany (*n* = 21), followed by Austria (*n* = 11) and Switzerland (*n* = 4). The number of subscales varied between one and 16 and the number of items per instrument ranged from two to 67. The development of 20 out of 31 identified instruments was based on other existing instruments available in English (e.g., translations of English original versions, see Additional file 3: File 5).

**Table 1:** Main characteristics of identified instruments

Instrument	Aims of the instrument	Number of subscales; number of items	Profession/role of participants	Characteristics of the intervention being assessed	Country
Hospital and health care setting					
AMMHTA Acceptance of mobile mental health treatment applications [53]	To assess the acceptance and intention to use mobile mental health treatment applications by young adults	7 subscales 33 items	(Potential) patients using mobile mental health treatment applications	Mobile mental health treatment applications	Germany
AGS Attitudes Towards Guidelines Scale [54]	To assess attitudes towards guidelines	7 subscales 14 items	Nurses working in an acute care hospital setting	Implementation of an evidence-based fall-prevention guideline into nursing practice in an acute care hospital setting	Austria
APOI-HP Attitudes towards Psychological Online Interventions – Health Professionals [34]	To assess psychotherapists’ acceptance of internet interventions	4 subscales 16 items	Psychotherapists with practice license	Web-based intervention to treat mild to moderate depression (EVIDENT trial)	Germany
APOI Attitudes towards Psychological Online Interventions [38]	To assess participants’ acceptance of internet interventions	4 subscales 16 items	Participants of a web-based intervention for treatment of depression	Web-based intervention to treat mild to moderate depression (EVIDENT trial)	Germany
CSQ-I Client Satisfaction Questionnaire adapted to Internet-based interventions [33, 58]	To assess global satisfaction with web-based interventions	1 scale 8 items	Participants of different interventions	Web-based psychosocial interventions (to prevent MDD, to treat vaginismus, or stress-management trainings)	Germany
CSQ-8 Client Satisfaction Questionnaire; [59, 63, 64]	Brief global measure to assess client satisfaction	1 scale 8 items	Patients receiving psychosocial or pain-related treatments	Satisfaction with different psychosocial or health-related treatments	Germany, Switzerland
CVF Competing Values Framework [55, 67]	To assess the organizational culture	4 subscales 25 items	Nurses working in an acute care hospital setting	Implementation of an evidence-based fall-prevention guideline into nursing practice in an acute care hospital	Austria
DTSQ(C) Diabetes Treatment Satisfaction Questionnaire (Change) [32, 56]	To assess diabetes treatment satisfaction sensitive to even small changes in satisfaction	1 scale 6 items	Patients relying on insulin therapy	Trainings for diabetes patients using different insulin treatments	Austria; Germany, Switzerland
DTSQ(S) Diabetes Treatment Satisfaction Questionnaire (Status) [32, 57]	To assess diabetes treatment satisfaction	1 scale 6 items	Patients relying on insulin therapy	Trainings for diabetes patients using different insulin treatments	Austria; Austria, Germany, Switzerland
EUUS Ease of Use and Usefulness Scale [47]	To assess the perceived usefulness, perceived ease of use and their intention for future teletreatment use	4 subscales 16 items	Patients participating in a myofeedback-based teletreatment	A myofeedback-based teletreatment for subjects with complaints in the neck and shoulder region	Germany, Belgium, the Netherlands, Sweden
EHRAS Electronic Health Record Acceptance Scale [41]	To identify the extent of acceptance of the electronic health record system and to examine influencing factors	8 subscales 30 items	General practitioners	Implementation of a nationwide electronic health record system for all patients	Austria
EGIP Evaluation of Guideline Implementation Process [55, 67]	To assess individuals’ perceptions of changes in relation to organizational changes	6 subscales 27 items	Nurses working in an acute care hospital setting	Implementation of an evidence-based fall-prevention guideline into nursing practice in an acute care hospital setting	Austria
FraSiK Frankfurt Patient Safety Climate Questionnaire for General Practices [49]	To assess safety culture (i.e., “an integrated pattern of individual and organizational behavior, based upon shared beliefs and values that continuously seeks to minimize patient harm”)	9 subscales 72 items in total, finally 47 items were used in factor analysis	Health care professionals working in general practices	Patient safety intervention in general practices	Germany
GQ-TPB Generic Questionnaire assessing “Theory of planned Behaviour” [30]	To assess physicians’ willingness to implement complex medical interventions and the factors influencing this willingness	2 subscales 41 items	General practitioners who received a training in arriba-lib	Arriba-lib, a multimodular electronic library of decision aids	Germany
GUQ-DUR German Utilization Questionnaire - Dissemination and Use of Research [50]	To measure attitude, availability, and support towards implementation of research in nursing practice	4 subscales original version: 47 items extended version: 58 items	Registered nurses (non-) participating in trainings in evidence-based nursing	Additional courses and training in evidence-based nursing to increase the research use in daily practice	Austria
HSOPSC Hospital Survey on Patient Safety Culture [43]	To assess safety climate from a staff perspective with 10 safety climate dimensions and 2 outcome dimensions	12 subscales 39 items	Clinicians and staff	A standardized team-training program for health care professionals (Zech et al., 2017)	Switzerland
KFPG Knowledge about fall prevention guideline [54]	To assess knowledge about the guideline, fall prevention, recommended intervention, and risk of falls	1 scale (7 single- and 6 multiple-choice items)	Nurses working in an acute care hospital setting	Implementation of an evidence-based fall-prevention guideline into nursing practice in an acute care hospital setting	Austria
OLS Organisational Learning Survey Instrument [55, 67]	To assess the organizational learning capability	5 subscales 21 items	Nurses working in an acute care hospital setting	Implementation of an evidence-based fall-prevention guideline into nursing practice in an acute care hospital setting	Austria
PEACS Patients’ Experiences Across Health Care Sectors [35]	Using a generic questionnaire to assess the experiences and reported outcomes in patients receiving treatment across a range of health care sectors	3 subscales for rating scales: 12 items 6 subscales for reporting items: 28 items	Patients having undergone another surgery or treatment in the past 12 months	Quality of care across different health care sectors	Germany
PUA-MSM Physicians’ usage and acceptance of different medication safety measures [42]	To measure user acceptance of a decision support system	12 subscales 27 items	Permanent emergency department staff in one hospital	Medication safety interventions such as repeated training, pocket checklists listing critical drugs and symptoms, computerized clinical decision support system	Germany
SAMS-P and SAMS-S Satisfaction with Medication Scale - Parents version (P) or Patients version (S) [51]	To assess the satisfaction with ADHD medication of parents and children in a post-marketing observational study	SAMS-P: one scale 12 items SAMS-S: 2 subscales 12 items	Children suffering from ADHD and receiving medication	Assessment of the effectiveness and safety of Equasym XL ®, a ADHD medication	Germany
SOAPC Survey of Organizational Attributes for Primary Care [31]	To assess organizational attributes and internal resources in general practices	4 subscales 23 items	Clinicians and staff	Protocol-based care management delivered by medical assistants [80]	Germany
USE Usefulness Scale for Patient Information Material [48]	To assess cognitive, emotional, and behavioral aspects of “subjective usefulness” of a patient information material	3 subscales (final version) 9 items	Clinical sample of patients with depression or patients with chronic low back pain	Written patient information material about either chronic low back pain or depression depending on the diagnosis	Germany
Education systems					
CtI Commitment to Innovation [52]	To assess the directors of child care centres commitment to innovation	1 scale 2 items	Early childhood educators in child care centers	School-specific compensatory education before entering the school system	Germany
SVS Social Validity Scale [36]	To assess the social validity of prevention programs (acceptance of programs, importance of prevention effects)	3 subscales 17 items	Primary school children and their parents	School-based program to foster social competence or prevent aggressive behavior	Germany
Workplace settings					
IOHORC Individual and organizational health-oriented readiness for change [45]	To assess the individual and organizational health-oriented readiness for change to target individuals’ behavior and the work environment	4 subscales 8 items	Participants of a stress management intervention	Comprehensive longitudinal stress management intervention	Switzerland
WHPCI Worksite Health Promotion Capacity Instrument [39]	To assess health promotion willingness of a company to implement worksite health promotion; to assess the extent of health promotion management	2 subscales 21 items	Company representatives (owner, managing director, head of division, …)	Worksite health promotion intervention	Germany
Multiple settings					
GSE General Self-Efficacy Scale [55, 65, 66]	To assess optimistic self-beliefs to cope with a variety of difficult demands in life	1 scale 10 items	Breimaier et al.: nurses working in an acute care hospital; Hinz et al.: general population	Implementation of an evidence-based fall-prevention guideline into nursing practice in an acute care hospital setting	Austria; Germany
GLTSI German version of the Learning Transfer Systems Inventory [37, 40, 60, 61]	To assess information about the creation of circumstances to foster the training transfer effects	Kauffeld et al. (2008) [37]: 16 subscales 67 items	Training participants	Trainings to improve work-related social skills or to increase empowerment	Germany
PKSMHP Perceived Knowledge of the Skills (PKS) needed in the area of mental health promotion (MHP) [46]	To assess the perceived need of knowledge and skills in the area of mental health promotion	3 subscales 9 items	Practitioners in different settings	Intervention to promote people’s mental health in different settings	Austria, Germany, Estonia, Finland, Ireland
SS-TC Short Scale - Technology [44, 62]	To assess technology commitment in terms of three facets: technology acceptance, technology competence, technology control	3 subscales 12 items	General population and specific older users of a new technology	Acceptance of seniors towards automatic in home fall detection devices or acceptance of mobility platform for older inhabitants	Germany

*Abbreviations*: *ADHD* attention deficit hyperactivity disorder, *MDD* major depressive disorder

Overall, we identified only six instruments where the assessment process was based on different samples [32, 33, 37, 40, 44, 55–61, 63, 64, 66], resulting in a more thorough assessment.

### Psychometric properties of the instruments

The amount and the quality of information offered for each instrument varied considerably. On average, 4.9 out of 12 psychometric criteria were reported per instrument, ranging from three to nine criteria. Only ten instruments conveyed information on six or more different psychometric criteria [30–33, 37, 40, 44, 48, 51, 55, 57–66]. All or most articles reported on *usability* (100%) and *internal consistency* (97%) of the scales (Table 2). In contrast, information on *construct* and *criterion validity* was rarely reported (6–16%). No instrument reported on the psychometric property *responsiveness.*

**Table 2:** Overview of psychometric properties of the instruments

Psychometric properties	*N*	%	M	SD	Md	min	max
Internal consistency	30	97	1.8	1.4	2.0	-1	4
Convergent validity	5	16	0.6	1.4	4.0	0	4
Discriminant validity	5	16	0.6	1.4	4.0	0	4
Known-groups validity	2	6	0.2	0.9	3.5	0	4
Predictive validity	2	6	0.1	0.2	1.0	0	1
Concurrent validity	10	32	0.5	1.0	2.0	-1	3
Structural validity	21	68	1.5	1.8	2.0	-1	4
Responsiveness	0	0	0.0	0.0	0.0	0	0
Norms	14	45	1.3	1.6	3.0	0	4
Usability	31	100	3.2	0.6	3.0	2	4
Test-retest reliability	3	10	0.1	0.3	1.0	0	1
Face and content validity	29	94	0.9	0.2	1.0	0	1

*Abbreviations*: *M* mean over all ratings, *max* maximum, *Md* median rating considering only those instruments which provided information on that aspect, *min* minimum, *n* number of instruments with a rating of −1, 1, 2, 3 or 4; %, percentage of instruments with a rating of −1, 1, 2, 3 or 4, *SD* standard deviation over all ratings

Explanation: This table displays the aggregated rating information for each psychometric property based on 31 identified instruments. Rating ranges from − 1 “poor”, 0 “no information”, 1 “minimal emerging”, 2 “adequate”, 3 “good”, 4 “excellent” for all the psychometric properties except test-retest reliability, and face and content validity where the rating was 0 “no information provided” and 1 “information provided”

The results for different settings can be found in Additional file 4.

**Fig. 2:**
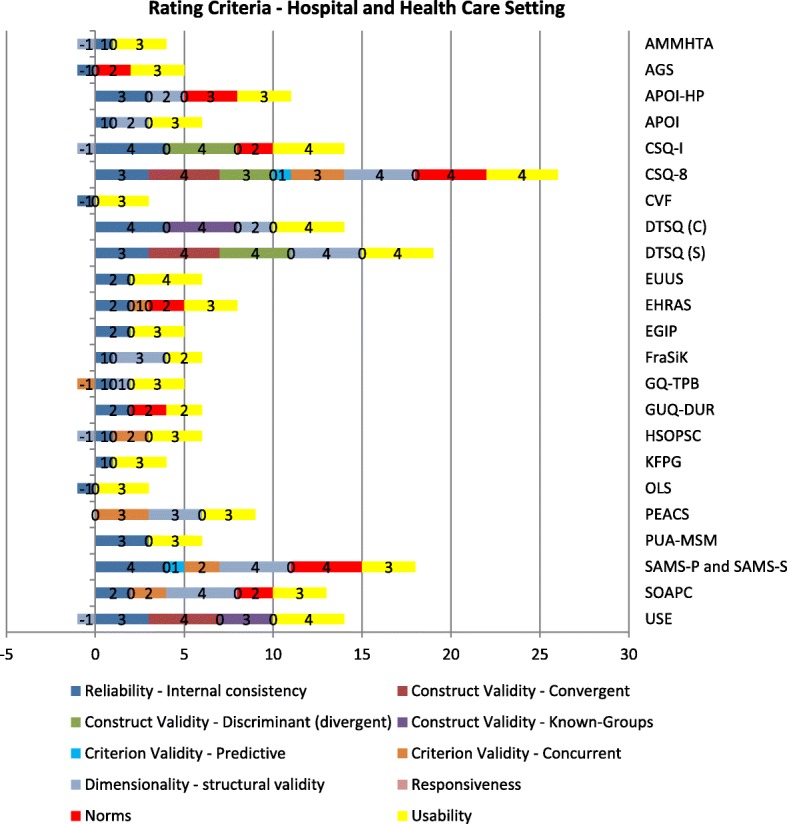
PAPERS rating criteria of instruments used in the hospital and health care setting

**Fig. 3:**
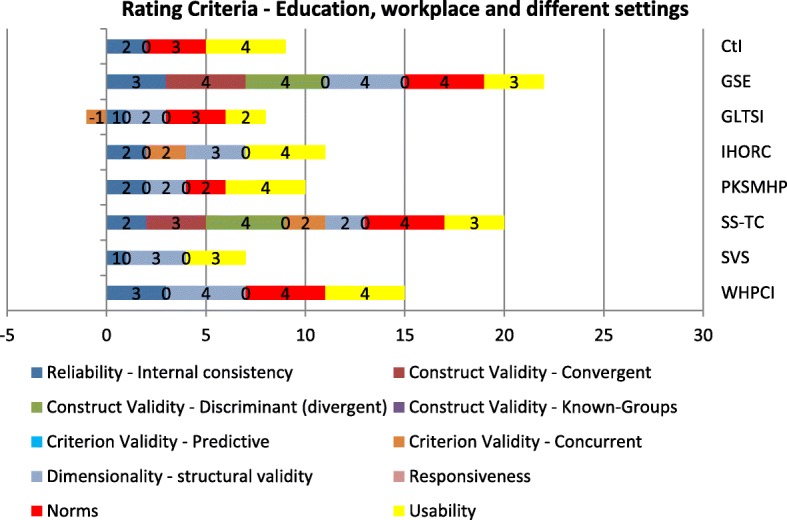
PAPERS rating criteria of instruments used in the education setting, workplace setting, and different settings

The specific results for the included instruments are depicted in Fig. 2 for the hospital and health care settings and in Fig. 3 for the educational, workplace, and diverse settings. In the following sections, each psychometric property is described separately.

#### Reliability—internal consistency

This criterion refers to the extent that items on a scale or subscale can be correlated to each other due to their assessment of the same construct. The Cronbach’s α coefficient is the most frequently used indicator [69]. Most instruments (30/31; 97%) reported data on *reliability* of either the total scale or subscales. On average, the rating was 1.8 (SD = 1.4), ranging from − 1 to 4. The median rating assigned to only those instruments, which provided information on that aspect, was 2.0 representing an “adequate” rating (Table 2). Nine instruments [31, 41, 44–47, 50, 52, 55, 62, 67] showed at least adequate Cronbach’s α values (0.70 < α ≤ 0.79). Only seven instruments received a good rating, *α* ≥ 0.80 [32, 34, 39, 42, 44, 48, 55, 57, 59, 63, 64, 66] and three an excellent rating, *α* ≥ 0.90 [32, 33, 51, 56, 58]. Cronbach’s α values per instrument are depicted in Additional file 3: File 1.

#### Construct validity—convergent, discriminant, and known-groups

This term describes the extent that a group of items characterize the construct to be measured [70]. While *convergent validity* is seen as the accordance in empirical relatedness of theoretically allied constructs, *discriminant validity* is seen as the empirical discordance of theoretically unrelated constructs [70]. *Known-groups validity* seeks to determine whether groups with distinct features can be differentiated by their responses on a new instrument [29, 70].

Overall, only about a quarter of the instruments (7/31; 23%) informed on at least one aspect of construct validity (Table 2). However, if any authors offered information on those aspects, the median ratings showed good or excellent results (range, 3.5–4 points). Four instruments (CSQ-8, DTSQ-S, GSE, and SS-TC) disclosed information on *convergent* and *discriminant validity* [32, 44, 55, 57, 59, 62–64, 66] and for one instrument each, information only on *convergent validity* [48] and *discriminant validity* [33, 58] was reported: the “Client Satisfaction Questionnaire-Internet” (CSQ-I) and the “Usefulness Scale for Patient Information Material” (USE), respectively. The median for instruments being tested for these validity aspects was 4.0 (Table 2). For two instruments [32, 48, 56], the authors reported on the assessment of *known-groups validity* (Table 2). The “Diabetes Treatment Questionnaire – Change” (DTQ-C) [32, 56] received a rating of “4—excellent” (i.e., two or more statistically significant differences between groups detected and hypotheses tested) and the USE [48] received a rating of “3—good” (i.e., one expected difference was shown between groups). Detailed information regarding construct validity can be found in Additional file 3: File 2.

#### Criterion validity—predictive and concurrent

This criterion refers to the extent to which a new instrument is correlated with a “gold standard” (i.e., measuring a distinct outcome). If an instrument is additionally administered at some point in the future, it refers to predictive validity. If it is administered at the same time, the validity aspect is called concurrent validity [69]. The CSQ-8 [59, 63, 64] and the SAMS-P/SAMS-S [51] reported data on both aspects, *predictive* and *concurrent validity*. Additionally, authors provided data on *concurrent validity* for eight other questionnaires [30, 31, 35, 37, 40, 41, 43–45, 60–62]. The median rating was 1.0 for predictive validity and 2.0 for concurrent validity (Table 2). The CSQ-8 [59, 63, 64] and the SAMS-P/SAMS-S [51] received only a rating of “1—minimal/emerging validity” for the predictive validity (i.e., Pearson’s *r* reached only a value between 0.10–0.29). Only two out of ten instruments including the CSQ-8 [59, 63, 64] and the “Patients’ Experiences Across Health Care Sectors” (PEACS) [35] verified a “3—good” concurrent validity (i.e., 0.50 < Pearson’s *r* ≤ 0.69; see Additional file 3: File 3).

#### Dimensionality—structural validity

This term is defined as the extent to which an instrument reveals the internal structure of its components as expected or theoretically hypothesized [69]. A prominent way to assess structural validity is via factor analysis. Authors of two thirds of the instruments (21/31, 68%) revealed information on aspects of *structural validity* [30–40, 43–46, 48, 49, 51, 53, 55–66]. Overall, the median rating for structural validity was 2.0, showing a wide variety, mirrored in the ratings, ranging from − 1 to 4 (Table 2). For example, the explained variance of the factor analyses stretched between 35% [38] and 75% [32, 56]. Six instruments including the CSQ-8 [59, 63, 64], the DTSQ-S [32, 57], the SAMS-P/SAMS-S [51], the “Survey of Organizational Attributes for Primary Care” (SOAPC) [31], the “Worksite Health Promotion Capacity Instrument” (WHPCI) [39], and the GSE [55, 65, 66] reached an excellent *structural validity* rating, as the explained variance was > 50% and the sample size was sufficiently large. The best rating (see Additional file 3: File 1) for the assessment of confirmatory factor analysis was “3—good,” which was awarded to two instruments: the “Social Validity Scale” (SVS) [36] and the “Individual and organizational health-oriented readiness for change questionnaire” (IOHORC) [45].

#### Norms

*Norms* in terms of central tendency and distribution of the total score [29] were available for about half (14/31, 45%) of the instruments [31, 33, 34, 37, 39–41, 44, 46, 50–52, 54, 55, 58–66]. The median for the rating of this dimension was “3—good,” ranging from 0 to 4 (Table 2). Age- and gender-specific norms (see Additional file 3: File 4) were only available for the GSE [65, 66].

#### Usability

This is a pragmatic criterion that refers to the ease of use in terms of the necessary number of items to measure a construct. This criterion was not included in the PAPERS criteria [29], but in the first rating scale version designed by Lewis and colleagues [21]. All instruments revealed information on usability. Ten instruments had fewer than ten items [32, 33, 39, 45–48, 52, 56–59, 63, 64], receiving a “4—excellent” rating, and 18 instruments had greater than ten but fewer than 50 items, receiving a “3—good” rating. The median rating was 3.0, ranging from 0 to 4 (Table 2). Clinton-McHarg and colleagues [19] also considered the number of missing items observed following instrument administration. Overall, eight instruments reported on the maximum value or range of missing values [30, 31, 35, 39, 41, 45, 46, 49]. The reported maximum percentage of missing values was 13.2% for a specific item in the “Perceived Knowledge of the Skills needed in the area of Mental Health Promotion scale” (PKSMHP) [46]. Detailed information can be found in Additional file 3: File 4.

#### Test-retest reliability

This criteria is defined as the stability of the instrument over time [70]. This aspect was not included in the PAPERS criteria [29]. Only three instruments reported on *test-retest reliability*: the “Generic Questionnaire assessing ‘Theory of planned Behaviour’” (GQ-TPB) [30], the PEACS [35], and the SAMS-P/SAMS-S [51]. Only the assessment study of GQ-TPB [30] applied the appropriate administration period of 2 to 14 days while the others [35, 51] relied on a longer administration period (3 to 10 weeks). None of the instruments received adequate *test-retest reliability* (*r* > 0.70) for all of the subscales. The test-retest coefficients ranged between 0.54 and 0.86 (see Additional file 3: File 3).

#### Face and content validity

*Face validity* refers to the extent researchers and those who complete an instrument agree that the instrument measures what it purports to measure [70]. *Content validity* refers to the instrument’s development process and considers selection of items, theory relatedness, and formal assessment of the instrument’s content [19]. Neither aspect was included in the PAPERS criteria [29]. Most of the instruments (94%) provided background on their instrument’s development process. Authors used theoretical knowledge in the development process of 19 instruments. To improve *face and content validity*, researchers of 15 instruments applied diverse methods such as expert ratings of the draft version, Delphi groups, pre-testing of instruments with the intended population, and cognitive pre-tests (see Additional file 3: File 5).

#### Responsiveness

This refers to the ability of an instrument to detect change over time [29, 71]. No instrument provided data on this dimension.

### Mapping against CFIR and IOF constructs

A total of 19 instruments included at least one of the 38 CFIR constructs (see Additional file 5). On average, each instrument assessed two constructs, ranging from one to seven constructs. The “German version of the Learning Transfer Systems Inventory” (GLTSI) [37, 40, 60, 61]  measured seven constructs. Overall, the different constructs were investigated rather unevenly. Two CFIR constructs, *networks & communications* [31, 37, 43, 49, 50, 55, 67] and *individual’s knowledge and beliefs about the intervention* [30, 37, 41, 44, 45, 54] were assessed six times, and the domain *leadership engagement* [37, 43, 49, 52, 55, 67] was operationalized five times. However, 22 constructs of the CFIR framework were not covered by instruments in German at all. The majority of those belonged to the CFIR domains intervention characteristics, outer setting, and process. The domain inner setting, however, was investigated intensively: 13 instruments covered the 14 CFIR constructs of that domain a total of 25 times.

Altogether, 17 instruments enabled users to assess at least one of IOF’s eight constructs. On average, one instrument enabled the testing of 1.4 IOF constructs. Overall, it ranged from one to three constructs [37, 40, 60, 61]. These instruments were the “Acceptance of Mobile Mental Health Treatment Applications scale” (AMMHTA) [53] and the “Attitudes towards Guidelines Scale” (AGS) [54]. The most frequently (*n* = 16) operationalized domain of IOF was *acceptability*, followed by *feasibility* (*n* = 4), *appropriateness* (*n* = 3), and *cost* (*n* = 1). No instrument covered the domains *adoption*, *fidelity*, *penetration*, and *sustainability*.

## Discussion

Currently, there is a lack of instruments available for assessing implementation processes in German-speaking countries. Several initiatives and reviews [19, 21] have recently been conducted to locate questionnaires that assessed contextual factors influencing implementation processes and outcomes. Nevertheless, only one questionnaire was identified that had been adapted for use in the German language. Hence, we conducted a systematic review to detect instruments used for measuring implementation constructs specifically in the German language. Overall, we identified 38 articles reporting on the psychometric properties of 31 instruments. While we could identify 23 different instruments for the hospital and health care setting, comparably fewer published instruments could be identified for other settings (e.g., workplace, community, education, and childcare settings). On average, each instrument provided information on 4.9 out of 12 psychometric criteria, ranging from three to nine. Generally, most articles provided information on the *internal consistency* (97%) but, authors rarely reported on *construct validity* (23%). The fact that validity aspects were not reported was reflected by other reviews in this area [19–21, 23]. The missing information on validity is significant as it is unclear whether or not the instruments are actually measuring what they intend to measure and if the conclusions based on this research are valid and meaningful.

Furthermore, the quality of information described for *reliability was* only “2—adequate”. Overall, these results show that the majority of the currently applied instruments require further refinement, more extensive item development, and retesting of scales. Without well-developed instruments, researchers will continue to use self-developed instruments, which will impair the ability of the implementation science community in German-speaking countries to further test theories and advance the field’s knowledge. When researchers use existing instruments with low validity and reliability, they should be aware that results have to be interpreted with caution and that they should use multiple sources for assessing implementation variables [72].

Some of the instruments showed reliable results, especially the ones assessing the IOF construct *acceptability*, such as the “Client Satisfaction Questionnaire” (CSQ-8) [59, 63, 64] and the “Diabetes Treatment Satisfaction Questionnaire - Status” (DTSQ-S) [32, 57]. The CSQ-8 received 26 out of 40 possible points and the DTSQ-S attained 19 points (Figs. 2 and 3): Two instruments, the “General Self-Efficacy Scale” (GSE) [55, 65, 66] and the “Short Scale – Technology Commitment” (SS-TC) [44, 62] used in settings other than in hospitals and health care facilities also showed a profound assessment of six different psychometric criteria, achieving 22 and 20 points, respectively.

Overall, the identified instruments contributed very unevenly to the 38 CFIR and eight IOF constructs. The questionnaires exposed here covered 20 out of 46 constructs of the aforementioned frameworks. Specifically, a serious shortage in instruments could be attributed to the CFIR domains *intervention characteristics*, *outer setting*, and *process* as well as the IOF constructs *adoption*, *fidelity*, *penetration*, and *sustainability*. While a review of instruments in the field of mental health [21] found a similar majority of instruments assessing *acceptability*, the high number of identified instruments in their review for the construct *adoption* in comparison to our review was surprising. This may be partly due to the different coding processes of the reviewers. Despite the high number of instruments assessing *acceptability* and *appropriateness*, instruments operationalizing these constructs in the public health and community settings or in a generic way were scarce. To foster the knowledge generation in that area, these instruments need to be developed. Furthermore, the CFIR subdomains *intervention characteristics*, *outer setting*, and *process* require future attention regarding the development process of instruments [19, 21]. Both reviews by the groups of Lewis and Clinton-McHarg [19, 21] mirrored the findings of the most frequently assessed domains being *inner setting* and *characteristics of individuals*.

In general, the overlap of identified instruments between our study and the aforementioned systematic reviews [19, 21] was rather minimal. The missing congruency might be attributed to the different foci and inclusion criteria of the reviews: Lewis and colleagues [21] focused on mental health interventions, while we did not include instruments assessing the day-to-day psychotherapeutic treatment. While Clinton-McHarg’s group [19] included only studies conducted in the public health sector assessing CFIR but not IOF constructs, our review included the general hospital and health care settings as well, where most instruments had been applied. Another difference between the previously conducted reviews and our work was that the former excluded studies not published in English [19, 21], and therefore, those instruments published in German were not included [52, 62, 63]. Clinton-McHarg et al. [19] showed that the majority of the instruments (38 out of 51) were developed in the USA, Canada, and other English-speaking countries, thereby revealing the prominent position of the English-speaking implementation science community. This has been reflected by our result that the development of 20 out of 31 identified instruments was based on other existing instruments available in English (e.g., translations of English original versions). And while both instruments were captured by the different searches and identification processes, some instruments in German [42, 50] were adaptations of the original versions in English [73, 74] and, therefore, were not explicitly listed in the aforementioned reviews and vice versa.

### Limitations

Despite a thoroughly developed and tested bibliographic search strategy, some relevant publications may have been missed. To combat potential drawbacks of our strategy, we extended our searches to include citation forward techniques and approached experts for suggestions of eligible articles [75]. Nevertheless, it is important to mention that we only used the defined source article by the SIRC review team for forward citation search, although often more than one reference was listed. If authors residing in German-speaking countries relied on another publication, we would not have been able to identify it. Similar to the approach by Clinton-McHarg and her group [19], we did not rely on gray literature searches, assuming that authors taking the thorough effort of developing or translating a well-designed instrument [69, 70, 76] would publish it in indexed journals. Furthermore, as we were interested in instruments which have already been used for the evaluation of an intervention, we did not include studies that covered CFIR constructs that had not been used in such an assessment process [77–79]. As mentioned above, a further limitation of the review was that the alignment of the identified scales and subscales to the CFIR and IOF constructs was done on scale but not item level. Some misclassifications may have happened as no clear and non-overlapping definitions of constructs are currently available [24].

Nevertheless, the present work provided an overview including an evaluation of the instruments’ psychometric properties of available German instruments used for assessing implementation constructs. This readily available information can guide future research efforts in this area. For existing instruments, it seems to be necessary to improve the internal consistency of the scales and to promote research on construct and criterion validity. Furthermore, the mapping process onto the CFIR and IOF constructs revealed that instruments assessing the CFIR domains intervention characteristics, outer setting, and process and the IOF domains adoption, fidelity, penetration, and sustainability are missing. In addition, one generic questionnaire measuring the most relevant IOF constructs including acceptability, appropriateness, and feasibility would advance the field.

## Conclusions

Some instruments (e.g., CSQ-8, DTSQ-S, GSE, and SS-TC) present a good starting point for assessing relevant CFIR and IOF constructs in the German language. Nevertheless, a continuous effort is needed for the improvement of existing instruments regarding the reliability and construct validity in particular, but also for the development of relevant missing instruments. This is especially significant for instruments in the public health and community settings. We encourage pooling the efforts in the German language implementation science community to prioritize which instruments should be developed or translated. In this way, German-speaking implementation researchers can foster a reliable and valid operationalization of implementation frameworks in multiple contexts while promoting an economically sensible use of research resources.

## Additional files

Additional file 1: Documentation search strategy. (DOCX 31 kb)

Additional file 2: Description rating criteria. (DOCX 43 kb)

Additional file 3: 1. Details psychometric criteria—reliability and structural validity. 2 Details psychometric criteria—construct validity. 3. Details psychometric criteria—criterion validity, test-retest reliability. 4. Details psychometric criteria—norms, usability. 5. Details psychometric criteria—face and content validity, responsiveness. (ZIP 158 kb)

Additional file 4: Overview of instrument’s psychometric properties used in settings. (DOCX 27 kb)

Additional file 5: Mapping CFIR and IOF constructs. (DOCX 66 kb)

## Abbreviations

ADHD: Attention deficit hyperactivity disorder
CFIR: Consolidated Framework for Implementation Research
EBP: Evidence-based practices
IOF: Implementation Outcomes Framework
M: Mean
Max: Maximum
Md: Median
MDD: Major depressive disorder
Min: Minimum
*n*: Number
PRISMA: Preferred Reporting Items for Systematic Reviews and Meta-Analyses
SD: Standard deviation
SIRC: Society for Implementation Research Collaboration

## Footnotes

1 We did not include the “other personal attributes” construct of the CFIR domain “characteristics of individuals” as no clear definition was available to guide a selection process.
