# Supplementary material for: German language questionnaires for assessing implementation constructs and outcomes of psychosocial and health-related interventions: a systematic review
Source: Implement Sci. 2018 Dec 12;13:150. doi: 10.1186/s13012-018-0837-3 (PMC6292038; doi:10.1186/s13012-018-0837-3)
Supplement: Supplementary file 1 — Documentation search strategy. (DOCX 31 kb) [file 13012_2018_837_MOESM1_ESM.docx]

**Additional File 1: Documentation Search Strategy**

# PubMed Search Strategy (August 23, 2017)

| **Search** | **Terms** | **Hits** |
| --- | --- | --- |
| [#1](https://www.ncbi.nlm.nih.gov/pubmed/advanced)4 | Search **(#9 AND #10 AND #5 AND #11 AND #8)** Sort by: **Relevance** Filters: **Publication date from 1984/12/31 to 2017/12/31; Humans** | [1082](https://www.ncbi.nlm.nih.gov/pubmed/?cmd=HistorySearch&querykey=15) |
| [#1](https://www.ncbi.nlm.nih.gov/pubmed/advanced)3 | Search **(#9 AND #10 AND #5 AND #11 AND #8)** Sort by: **Relevance** Filters: **Humans** | [1082](https://www.ncbi.nlm.nih.gov/pubmed/?cmd=HistorySearch&querykey=14) |
| [#1](https://www.ncbi.nlm.nih.gov/pubmed/advanced)2 | Search **(#9 AND #10 AND #5 AND #11 AND #8)** | [1376](https://www.ncbi.nlm.nih.gov/pubmed/?cmd=HistorySearch&querykey=13) |
| [#1](https://www.ncbi.nlm.nih.gov/pubmed/advanced)1 | Search **(#6 OR #7)** | [10517598](https://www.ncbi.nlm.nih.gov/pubmed/?cmd=HistorySearch&querykey=12) |
| [#10](https://www.ncbi.nlm.nih.gov/pubmed/advanced) | Search **(#3 OR #4)** | [459909](https://www.ncbi.nlm.nih.gov/pubmed/?cmd=HistorySearch&querykey=11) |
| #9 | Search **(#1 OR #2)** | [5529460](https://www.ncbi.nlm.nih.gov/pubmed/?cmd=HistorySearch&querykey=10) |
| [#](https://www.ncbi.nlm.nih.gov/pubmed/advanced)8 | Search **(Austria[Affiliation] OR Germany[Affiliation] OR Switzerland[Affiliation] OR Österreich[Affiliation] OR Oesterreich[Affiliation] OR Deutschland[Affiliation] OR Schweiz[Affiliation])** | [1133606](https://www.ncbi.nlm.nih.gov/pubmed/?cmd=HistorySearch&querykey=9) |
| [#](https://www.ncbi.nlm.nih.gov/pubmed/advanced)7 | Search **"program development"[MeSH Terms]** | [25813](https://www.ncbi.nlm.nih.gov/pubmed/?cmd=HistorySearch&querykey=8) |
| #[6](https://www.ncbi.nlm.nih.gov/pubmed/advanced) | Search **(intervention OR treatment OR practice OR guideline OR innovat* OR "health promotion" OR prevention)** | [10510385](https://www.ncbi.nlm.nih.gov/pubmed/?cmd=HistorySearch&querykey=7) |
| [#5](https://www.ncbi.nlm.nih.gov/pubmed/advanced) | Search **(implement* OR change OR adopt OR sustain OR disseminat* OR "quality improv*" OR diffus*)** | [1769698](https://www.ncbi.nlm.nih.gov/pubmed/?cmd=HistorySearch&querykey=5) |
| [#4](https://www.ncbi.nlm.nih.gov/pubmed/advanced) | Search **("validation studies as topic" OR psychometrics[MeSH Terms])** | [65718](https://www.ncbi.nlm.nih.gov/pubmed/?cmd=HistorySearch&querykey=4) |
| [#3](https://www.ncbi.nlm.nih.gov/pubmed/advanced) | Search **(psychometrics OR reliability OR validity OR acceptability OR validation)** | [459909](https://www.ncbi.nlm.nih.gov/pubmed/?cmd=HistorySearch&querykey=3) |
| [#2](https://www.ncbi.nlm.nih.gov/pubmed/advanced) | Search **("surveys and questionnaires"[MeSH Terms])** | [835716](https://www.ncbi.nlm.nih.gov/pubmed/?cmd=HistorySearch&querykey=2) |
| [#1](https://www.ncbi.nlm.nih.gov/pubmed/advanced) | Search **(questionnaire OR measure OR scale OR tool OR instrument OR survey OR assess* OR inventory)** | [5529460](https://www.ncbi.nlm.nih.gov/pubmed/?cmd=HistorySearch&querykey=1) |

# PsychInfo (via Ovid) Search Strategy (August 24, 2017)

| **Search** | **Terms** | **Hits** |
| --- | --- | --- |
| #1 | surveys/ or questionnaires/ | 23698 |
| #2 | psychometrics/ or classical test theory/ | 52417 |
| #3 | program development/ or program evaluation/ | 17174 |
| #4 | test reliability/ | 47930 |
| #5 | test validity/ | 67371 |
| #6 | (scale or questionnaire or measure or tool or instrument or survey or inventory or assess*).ab. or (scale or questionnaire or measure or tool or instrument or survey or inventory or assess*).ti. or (scale or questionnaire or measure or tool or instrument or survey or inventory or assess*).sh. or (scale or questionnaire or measure or tool or instrument or survey or inventory or assess*).id. | 1209879 |
| #7 | (psychometrics or reliability or validity or acceptability or validation).ab. or (psychometrics or reliability or validity or acceptability or validation).ti. or (psychometrics or reliability or validity or acceptability or validation).sh. or (psychometrics or reliability or validity or acceptability or validation).id. | 217514 |
| #8 | (implement* or change or adopt or sustain or disseminat* or "quality improv*" or diffus).ab. or (implement* or change or adopt or sustain or disseminat* or "quality improv*" or diffus).ti. or (implement* or change or adopt or sustain or disseminat* or "quality improv*" or diffus).sh. or (implement* or change or adopt or sustain or disseminat* or "quality improv*" or diffus).id. | 430288 |
| #9 | (intervention or treatment or practice or guideline or innovat* or prevention or "health promotion").ab. or (intervention or treatment or practice or guideline or innovat* or prevention or "health promotion").ti. or (intervention or treatment or practice or guideline or innovat* or prevention or "health promotion").sh. or (intervention or treatment or practice or guideline or innovat* or prevention or "health promotion").id. | 1032224 |
| #10 | 1 or 6 | 1210939 |
| #11 | 2 or 4 or 5 or 7 | 221456 |
| #12 | 3 or 9 | 1039458 |
| #13 | 8 and 10 and 11 and 12 | 6331 |
| #14 | (Austria or Germany or Switzerland or Oesterreich or Deutschland or Schweiz or German or deutsch*).in. or (Austria or Germany or Switzerland or Oesterreich or Deutschland or Schweiz or German or deutsch*).lg. or (Austria or Germany or Switzerland or Oesterreich or Deutschland or Schweiz or German or deutsch*).ab. | 260584 |
| #15 | 13 and 14 | 345 |

# PSYNDEXplus Literature and Audiovisual Media 1977 to July 2017 and PSYNDEXplus Tests 1945 to May 2017 (via Ovid) Search Strategy (August 24, 2017)

| **Search** | **Terms** | **Hits** |
| --- | --- | --- |
| #1 | surveys/ | 1489 |
| #2 | questionnaires/ | 9588 |
| #3 | psychometrics/or classical test theory/ | 6592 |
| #4 | program development/ or program evaluation/ | 1675 |
| #5 | test reliability/ | 3757 |
| #6 | test validity/ | 6350 |
| #7 | (scale or questionnaire or measure or tool or instrument or survey or inventory or assess*).ab. or (scale or questionnaire or measure or tool or instrument or survey or inventory or assess*).ti. or (scale or questionnaire or measure or tool or instrument or survey or inventory or assess*).kp. or (scale or questionnaire or measure or tool or instrument or survey or inventory or assess*).sh. | 62903 |
| #8 | (psychometrics or reliability or validity or acceptability or validation).ab. or (psychometrics or reliability or validity or acceptability or validation).ti. or (psychometrics or reliability or validity or acceptability or validation).kp. or (psychometrics or reliability or validity or acceptability or validation).sh. | 13547 |
| #9 | (implement* or change or adopt or sustain or disseminat* or “quality improve*” or diffus*).ab. or (implement* or change or adopt or sustain or disseminat* or “quality improve*” or diffus*).ti. or (implement* or change or adopt or sustain or disseminat* or “quality improve*” or diffus*).kp. or (implement* or change or adopt or sustain or disseminat* or “quality improve*” or diffus*).sh. | 20499 |
| #10 | (intervention or treatment or practice or guideline or innovate* or prevention or “health promotion”).ab. or (intervention or treatment or practice or guideline or innovate* or prevention or “health promotion”).ti. or (intervention or treatment or practice or guideline or innovate* or prevention or “health promotion”).kp. or (intervention or treatment or practice or guideline or innovate* or prevention or “health promotion”).sh. | 78458 |
| #11 | (Austria or Germany or Switzerland or Oesterreich or Deutschland or Schweiz or German or deutsch*).in. or (Austria or Germany or Switzerland or Oesterreich or Deutschland or Schweiz or German or deutsch*).lg. or (Austria or Germany or Switzerland or Oesterreich or Deutschland or Schweiz or German or deutsch*).ab. | 331052 |
| #12 | 1 or 2 or 7 | 65286 |
| #13 | 3 or 5 or 6 or 8 | 20430 |
| #14 | 4 or 10 | 79080 |
| #15 | 9 and 12 and 13 and 14 | 280 |
| #16 | 11 and 15 | 264 |

# ERIC Search Strategy (August 23, 2017)

| **Search** | **Terms** | **Hits** |
| --- | --- | --- |
| S1 | (psychometrics OR reliability OR validity OR acceptability OR validation) AND (implement* OR change OR adopt OR sustain OR disseminat* OR improv* OR diffus*) AND (intervention OR treatment OR practice OF guideline OR innovat* OR promotion OR prevention) AND ((questionnaire OR measure OR scale OR tool OR instrument OR survey OR assess* OR inventory) OR SU.Exact("Surveys" OR "Questionnaires")) OR (Austria OR Germany OR Switzerland OR Österreich OR Oesterreich OR Deutschland OR Schweiz OR Austrian OR German OR Swiss) | 17 |
| Limitations | With the following limitations: peer-reviewed articles (journal articles, tests/questionnaires), Published after 31.12.1984 | 17 |
