# Supplementary material for: German language questionnaires for assessing implementation constructs and outcomes of psychosocial and health-related interventions: a systematic review
Source: Implement Sci. 2018 Dec 12;13:150. doi: 10.1186/s13012-018-0837-3 (PMC6292038; doi:10.1186/s13012-018-0837-3)
Supplement: Supplementary file 2 — Description rating criteria. (DOCX 43 kb) [file 13012_2018_837_MOESM2_ESM.docx]

**Additional File 2: Rating Criteria** Rating information adapted from Lewis and colleagues [29]

| Internal consistency | | Internal consistency refers to the extent the items of a scale or subscale are correlated. Cronbach’s α coefficient is the most frequently used indicator [69] |
| --- | --- | --- |
|  |  | "Note: If scale and subscale α values are given, provide all and apply 'worst score counts' rule." |
|  | -1 | Poor (p): Cronbach's α values of <0.50 |
|  | 0 | None (N): Internal consistency measures are not applicable for this measure OR classical test theory anchors are not appropriate, results reported using item response theory OR α values are not yet available for the full measure scale or any associated subscales. |
|  | 1 | Minimal/emerging (M): α values of = 0.50-0.69 |
|  | 2 | Adequate (A): α values of 0.70-0.79 |
|  | 3 | Good: (G): α values of 0.80-0.89 |
|  | 4 | Excellent: (E): α values ≥ 0.90 |
| Construct Validity - Convergent | | Convergent construct validity refers to the extent a new instrument which is theoretically related with another one, is in fact also related empirically [29, 71] |
|  |  | NOTE: If Perason's *r* is given, use the effect size calculator to calculate Cohen's d.  https://www.polyu.edu.hk/mm/effectsizefaqs/calculator/calculator.html  Also, note that these criteria also apply to comparisons between subscales. Also, note if one study provides more than one result on convergent construct validity, the mode score (i.e. the value which was achieved most often) should be taken. If two values were achieved in the same extent, then the lower value was taken. The "worst score counts" approach was applied to achieve a single rating between more than one study. |
|  | -1 | Poor (P): Cohens's *d* ≤ 0.10 |
|  | 0 | None (N): convergent validity measures are not applicable for this instrument OR not yet tested |
|  | 1 | Minimal/emerging (M): 0.10 < Cohen's *d* ≤ 0.20 |
|  | 2 | Adequate (A): 0.20 < Cohen's *d* ≤ 0.50 |
|  | 3 | Good: (G): 0.50 < Cohen's d ≤ 0.80 |
|  | 4 | Excellent (E): Cohen's d ≥ 0.80 |

| Construct Validity - Discriminant | | Discriminant validity refers to the extent a new instrument which is supposed to be theoretically distinct from another construct, is in fact also distinct [29] citing [71]. |
| --- | --- | --- |
|  |  | NOTE: If Perason's *r* is given, use the effect size calculator to calculate Cohen's d.  https://www.polyu.edu.hk/mm/effectsizefaqs/calculator/calculator.html  Also, note that these criteria also apply to comparisons between subscales. Also, note if one study provides more than one result on discriminant construct validity, the mode score (i.e. the value which was achieved most often) should be taken. If two values were achieved in the same extent, then the lower value was taken. The "worst score counts" approach was applied to achieve a single rating between more than one study. |
|  | -1 | Poor (P): Cohens's *d* > 0.80 |
|  | 0 | None (N): discriminant validity measures are not applicable for this instrument OR not yet tested |
|  | 1 | Minimal/emerging (M): 0.50 < Cohen's *d* ≤ 0.80 |
|  | 2 | Adequate (A): 0.20 < Cohen's *d* ≤ 0.50 |
|  | 3 | Good: (G): 0.10 < Cohen's d ≤ 0.20 |
|  | 4 | Excellent (E): Cohen's d ≤ 0.10 |
| Construct Validity - Known-Groups | | Know-groups validity seeks to determine whether groups with distinct features can be differentiated by their responses on a new instrument [29, 70]. |
|  |  | NOTE: relevant categories are: demographics, roles/professions, programs/ treatments, organizations, intervention conditions. Also, note if one study provides more than one result on known-groups validity, the mode score (i.e. the value which was achieved most often) should be taken. If two values were achieved in the same extent, then the lower value was taken. The "worst score counts" approach was applied to achieve a single rating between more than one study. |
|  | -1 | Poor (P): Known-Groups validity failed to be detected |
|  | 0 | None (N): Known-Groups validity not yet tested |
|  | 1 | Minimal/emerging (M): statistically significant difference between groups detected, but no hypothesis tested |
|  | 2 | Adequate (A): two or more statistically significant difference between groups detected, but no hypothesis tested |
|  | 3 | Good: (G): statistically significant difference between groups detected AND hypothesis tested |
|  | 4 | Excellent (E): two or more statistically significant differences between groups detected AND hypotheses tested |

| Criterion Validity - Predictive | | Predictive criterion validity refers to the extent (Pearson's r) a new instrument is correlated with a "gold standard" (measuring a distinct outcome) administered at some point in the future [69]. |
| --- | --- | --- |
|  |  | NOTE: If unstandardized regression coefficients (betas) are reported, use the effect size calculator to translate them into Pearon's *r* values and follow the same rules as above.  https://www.campbellcollaboration.org/escalc/html/EffectSizeCalculator-R7.php  If discriminant function analysis is reported, use the measure of variance explained. Anchors for this can be found in "Structural Validity" section. Also note, if multiple indices are given and they fall within differing rating anchors, use the mode score. If two numbers appear according to the worst score counts approach, the smaller rating is taken. |
|  | -1 | Poor (P): Pearson's *r* < 0.10 |
|  | 0 | None (N): convergent validity measures are not applicable for this instrument OR not yet tested |
|  | 1 | Minimal/emerging (M): 0.10 ≤ Pearson's *r <* 0.30 |
|  | 2 | Adequate (A): 0.30 ≤ Pearson's *r* < 0.50 |
|  | 3 | Good: (G): 0.50 ≤ Pearson's *r* < 0.70 |
|  | 4 | Excellent (E): Pearson's *r* ≥ 0.70 |
| Criterion Validity - Concurrent | | Concurrent criterion validity refers to the extent (Pearson's r) a new instrument is correlated with a "gold standard" (measuring a distinct construct of interest or outcome) administered at the same point in time [69]. |
|  |  | NOTE: If unstandardized regression coefficients (betas) are reported, use the effect size calculator to translate them into Pearon's *r* values and follow the same rules as above.  https://www.campbellcollaboration.org/escalc/html/EffectSizeCalculator-R7.php  If discriminant function analysis is reported, use the measure of variance explained. Anchors for this can be found in "Structural Validity" section. Also note, if multiple indices are given and they fall within differing rating anchors, use the mode score (i.e. the number which is repeated more often than any other). If two numbers appear according to the worst score counts approach, the smaller rating is taken. |
|  | -1 | Poor (P): Pearson's *r* < 0.10 |
|  | 0 | None (N): concurrent validity measures are not applicable for this instrument OR not yet tested |
|  | 1 | Minimal/emerging (M): 0.10 ≤ Pearson's *r <* 0.30 |
|  | 2 | Adequate (A): 0.30 ≤ Pearson's *r* < 0.50 |
|  | 3 | Good: (G): 0.50 ≤ Pearson's *r* < 0.70 |
|  | 4 | Excellent (E): Pearson's *r* ≥ 0.70 |
| Dimensionality - Structural Validity | | Structural validity is the extent to which an instrument reveals the internal structure of its components as expected or theoretically hypothesized [69]. A prominent way to assess construct validity is via factor analyses. |
|  |  | Abbreviations: CFI = Comparative Fit Index; GFI = Goodness of Fit Index; IFI = Incremental Fit Index; NFI = Normed Fit Index; RNI = Relative Noncentrality Fit Index; TLI = Tucker-Lewis Index;  RMSEA = Root Mean Square Error of Approximation; SRMS = Standardized Root Mean Residual; WRMR = Weighted Root Mean Residual; |
|  |  | NOTE: If multiple indices are given and they fall within differing rating anchors, use the mode score. If two numbers appear according to the worst score counts approach, the smaller rating is taken. |
|  | -1 | Poor (P): The sample consisted of less than 5 times the number of items AND exploratory factor analysis explained <25% of variance OR |
|  |  | CFI OR GFI OR IFI OR NFI OR RNI OR TLI ≤ 0.88 OR RMSEA OR SRMR ≥ 0.10 OR WRMR ≥ 0.92 |
|  | 0 | None (N): no exploratory or confirmatory analysis has yet been performed nor any Item Response Theory tests of (uni-)dimensionality have been conducted, or percent variance explained is reported OR analysis has been conducted but percent variance is unexplained and cannot be calculated OR only principal components analysis has been conducted |
|  | 1 | Minimal/emerging (M): the sample consisted of less than five times the number of items and an exploratory factor analysis explained less than 25% of the variance OR |
|  |  | CFI OR GFI OR IFI OR NFI OR RNI OR TLI = 0.88 < X ≤ to 0.90 OR RMSEA OR SRMR = 0.08 ≤ X < 0.10 OR WRMR = 0.90 ≤ X < 0.92 |
|  | 2 | Adequate (A): the sample consisted of less than five times the number of items but is less than 100 in total and an exploratory factor analysis explained less than 50% of the variance |
|  |  | CFI OR GFI OR IFI OR NFI OR RNI OR TLI = 0.90 < X ≤ to 0.95 OR RMSEA OR SRMR = 0.05 ≤ X < 0.08 OR WRMR = 0.85 ≤ X < 0.90 |
|  | 3 | Good: (G): the sample consisted of five times the number of items and is greater than or equal to 100 in total or the sample consisted of five to seven times the number of items but is less than 100 in total and an exploratory factor analysis explained less than 50% of the variance |
|  |  | CFI OR GFI OR IFI OR NFI OR RNI OR TLI = 0.95 < X ≤ to 0.97 OR RMSEA OR SRMR = 0.03 ≤ X < 0.05 OR WRMR = 0.83 ≤ X < 0.85 |
|  | 4 | Excellent: (E): the sample consisted of seven times the number of items and is greater than 100 in total and an exploratory analysis explained more than 50% of the variance |
|  |  | CFI OR GFI OR IFI OR NFI OR RNI OR TLI > 0.97 OR RMSEA OR SRMR ≤ X < 0.03 OR WRMR < 0.83 |
| Responsiveness | | Responsiveness refers to the characteristic of a measure to being able to detect change over time [29] citing [71]. |
|  |  | Abbreviations: SRM = standardized response mean |
|  | -1 | Poor (P): SRM < 0.10 OR Perason's *r* < 0.10 |
|  | 0 | None (N): the measure has either not been administered both pre- and post-implementation to evaluate sensitivity to change |
|  | 1 | Minimal/emerging (M): SRM = 0.10 ≤ X < 0.20 OR Pearson's *r* = 0.10 ≤ X < 0.30 |
|  | 2 | Adequate (A): SRM = 0.20 ≤ X < 0.50 OR Pearson's *r* = 0.30 ≤ X < 0.50 |
|  | 3 | Good (G): SRM = 0.50 ≤ X < 0.80 OR Pearson's r = 0.50 ≤ X < 0.70 |
|  | 4 | Excellent (E): SRM > 0.80 OR Pearson's r > 0.70 |
| Usability (instrument length) | | Usability is a pragmatic criterion and it refers to the ease of use related to the necessary number of items to measure a construct. (This criterion was not included in the PAPERS criteria). |
|  | 0 | None (N): the measure is not in the public domain |
|  | 1 | Minimal/emerging (M): the measure has more than 100 items |
|  | 2 | Adequate (A): the measure has more than 50 items but fewer than 100 items |
|  | 3 | Good (G): the measure has more than 10 items but fewer than 50 items |
|  | 4 | Excellent (E): the measure has fewer than 10 items |
| Norms | | Norms are defined as a description of the achieved results after an application of the instrument to a specific sample (mean, standard deviation, sample size) [69]. |
|  | -1 | Poor (P): Measures of central tendency and distribution for the total score (and subscales if relevant) based only on a very small (n > 50) sample are available |
|  | 0 | None (N): norms are not yet available |
|  | 1 | Minimal/emerging (M):) norms based only on a small (n = 50-99) sample are available |
|  | 2 | Adequate (A): norms based on a moderate (n = 100-299) sample are available |
|  | 3 | Good (G): norms based on a medium (n = 300-499) sample are available |
|  | 4 | Excellent (E): norms based on a large (n ≥ 500) sample are available |

Except otherwise stated via providing a reference, the rating system is taken from Lewis et al. [29].
