# Supplementary material for: German language questionnaires for assessing implementation constructs and outcomes of psychosocial and health-related interventions: a systematic review
Source: Implement Sci. 2018 Dec 12;13:150. doi: 10.1186/s13012-018-0837-3 (PMC6292038; doi:10.1186/s13012-018-0837-3)
Supplement: Supplementary file 3 — 1. Details psychometric criteria—reliability and structural validity. 2 Details psychometric criteria—construct validity. 3. Details psychometric criteria—criterion validity, test-retest reliability. 4. Details psychometric criteria—norms, usability. 5. Details psychometric criteria—face and content validity, responsiveness. (ZIP 158 kb) [file 13012_2018_837_MOESM3_ESM.zip › SIID_Additional File 3.1_PC_Reliability_Structural ValR1.docx]

**Additional File 3.1:** Reliability (internal consistency) and dimensionality (structural validity)

| **Instrument** | **Reliability – Internal Consistency** | | **Dimensionality – Structural Validity** | | | |
| --- | --- | --- | --- | --- | --- | --- |
|  | Subscale Name and α-value | Rating | Relation Sample Size:  Number of Items | Exploratory FA:  Explained Variance | Confirmatory FA | Rating |
| **Hospital and Health Care Setting** | |  |  |  |  |  |
| AMMHTA (53) | Task-technology fit: α=0.884 Social influence: α=0.647 Ease of use: α=0.700 Perceived usefulness: α=0.884 Trust: α=0.722 Self-efficacy: α=0.758 Intention to use: α=0.835 | 1 | Sample size: 125 33 items | NR | RMSEA: 0.096 CFI: 0.768 | -1 |
| AGS (54) | Overall T1: α=0.733 General attitude: α=0.719 Usefulness: α=0.551 Reliability: α=0.453 Lack of individual or team competence: α=0.135 Lack of organisational competence: α=0.509 Impracticality: α=0.702 Availability: α=0.392 | -1 | Sample size: 106 14 items | NR | NR | 0 |
| APOI-HP (34) | Overall scale α=0.83 Subscale α NR | 3 | Sample size: 428 16 items | NR | CFI=0.932 RMSEA=0.053 | 2 |
| APOI (38) | Overall scale α=0.77 Scepticism and perception of risks (SCE): α=0.62 Anonymity benefits (ABE): α=0.62 Technologization threat (TET): α=0.64 Confidence in effectiveness (CON): α=0.72 | 1 | Sample size for validation: 305 16 items | Explained variance: 35% | CFI=0.994 RMSEA=0.013 | 2 |
| CSQ-I (33, 58) | CSQ-I - 8-item scale:  α=0.97; omega 0.93-0.95 | 4 | Relation: 9.63 - 21.75 | NR | Study 1:  CFI=0.96 RMSEA=0.1 (p=0.002) Study 2: CFI=0.95 RMSEA=0.1 (p=0.02) | -1 |
| CSQ-8 (59, 63, 64) | CSQ-8: α=0.87-0.90 | 3 | Relation: 12.25-6647.13 | Explained variance: 53%-69% | NR | 4 |
| CVF (55, 67) | Overall: α=0.721 Group culture: α=0.81 Developmental culture: α=0.53 Hierarchical culture: α=0.47 Rational culture: α=0.37 | -1 | Sample size: 106 20 items | NR | NR | 0 |
| DTSQ(C) (32, 56) | DTSQ(C) - 6-item scale:  α=0.93 - 0.94 | 4 | Relation: 3.75 - 32.75 | DTSQ (C) - 6-items:  1 factor: 75.4% 18-item-scale:  2 factors: 58.3% and 17.1% | NR | 2 |
| DTSQ(S) (32, 67) | DTSQ(S) - 6-item scale:  α=0.85 - 0.86 | 3 | Relation: 9.5 - 53.0 | DTSQ(S) - 18-items: 4 factors explaining 69.8% of the variance | NR | 4 |
| EUUS (47) | Overall: α-values not reported  Ease of use: α=0.7 Perceived usefulness: α=0.8 | 2 | Sample size: 45 8 items | NR | NR | 0 |
| EHRAS (41) | Privacy concern: α=0.76 Social influence: α=0.90 Cost reduction: α=0.87 Improvement: α=0.91 Attitude: α=0.90 Intention to use: α=0.92 | 2 | Sample size: 204 28 items | NR | NR | 0 |
| EGIP (55, 67) | Overall: α=NR Characteristics of the change process: α=0.88 Extent of work unit change: α=0.90 Consequence of guideline implementation: α=0.84 Individual job impact: α=0.90 Demands-abilities: α=0.70 Values congruence: α=0.87 | 2 | Sample size: 106 27 items | NR | NR | 0 |
| FraSiK (49) | Reported for 9 subscales;  Overall: NR Teamwork climate: α=0.905 Error management: α=0.798 Safety of clinical processes: α=0.674 Perception of causes of errors: α=0.71 Job satisfaction: α=0.768 Safety of practice structure: α=0.552 Receptiveness to healthcare assistants and patients: α=0.639 Subsamples: Staff perception of management: α=0.753 Quality and safety of medical care: α=0.563 | 1 | Sample size: 332 72 items in total, but only 46 items appropriate for factor analysis (although 47 items are used in factor analysis) | Explained variance: 49.7% with 7 factors and two factors were analysed and reported separately; Originally proposed are 9 factors | NR | 3 |
| GQ-TPB (30) | Attitude: t0: α=0.87; t1: α=0.90 Subjective norm and perceived Behaviour control: t0: α=0.65; t1: α=0.60 | 1 | Sample size: 181 41 items | NR | SRMR= 0.08 RMR=0.04 GFI=0.92 AGFI=0.92 | 1 |
| GUQ-DUR (50) | Overall: NR Use: α=0.91  Attitude: α=0.90 (0.92 after correction) Availability: α=0.75 (0.82 after correction) Support: NR | 2 | Sample size: 178 47 items (translation of english version) 58 items (with additional items) | NR | NR | 0 |

| **Instrument** | **Reliability – Internal Consistency** | | **Dimensionality – Structural Validity** | | | |
| --- | --- | --- | --- | --- | --- | --- |
|  | Subscale Name and α-value | Rating | Relation Sample Size:  Number of Items | Exploratory FA:  Explained Variance | Confirmatory FA | Rating |
| HSOPSC (43) | Supervisor, manager expectations and actions promoting safety: α=0.78 Organisational learning - continuous improvement: α=0.68  Teamwork within units: α=0.73 Communication openness: α=0.64 Feedback and communication about error: α=0.79 Nonpunitive response to error: α=0.71 Staffing: α=0.61 Hospital management support for patient safety: α=0.83 Teamwork across hospital units: α=0.76 Hospital handoffs and transitions: α=0.71 Overall perceptions of safety: α=0.75 Frequency of event reporting: α=0.88 | 1 | Sample size: 568 39 items | Explained variance: 59.8% with eight factors; originally proposed are 12 factors | GFI (Global-fit-index): 0.878 NFI=0.859 TLI=0.901 RMSEA: 0.047 | -1 |
| KFPG (54) | Overall T1: α=0.69 | 1 | Sample size: 106 13 items | NR | NR | 0 |
| OLS (55, 67) | Overall: α=0.831 Clarity of purpose and mission: α=0.31 Leadership commitment and empowerment: α=0.75 Experimentation and rewards: α=0.74 Transfer of knowledge: α=0.59 Teamwork and group problem solving: α=0.55 | -1 | Sample size: 106 21 items | NR | NR | 0 |
| PEACS (35) | NR | 0 | Sample size for validation: 474 75 items in pilot version | Explained variance: 64% for 28 reporting items; 6 factors; | NR | 3 |
| PUA-MSM (42) | Perceived usefulness: α=0.88 Perceived ease of use: α=0.95 Image: α=0.88 Job relevance: α=0.87 Result demonstrability: α=0.86 Resistance to change: α=0.86 Intention to use: α=1.00 | 3 | Sample size: 9 27 items | NR | NR | 0 |
| SAMS-P and SAMS-S (51) | SAMS-P: Overall: α=0.96  SAMS-S: Overall: α=0.92 | 4 | Sample size Parents: 589 Sample size Patients: 552 12 items (each version) | SAMS-P: Explained variance: 71.3% with 1 factor SAMS-S: Explained variance: 66.9% with 2 factors | NR | 4 |
| SOAPC (31) | Overall SOAPC score: α=0.84 Communication: α=0.76 Decision making: α=0.82 Stress/chaos: α=0.81 History of change: α=0.70 | 2 | Sample size: 297 21 items | Explained variance: 55.9% with four factors | NR | 4 |
| USE (48) | Overall: α=0.94 Cognitive: α=0.84 Emotional: α=0.94 Behavioural: α=0.91 | 3 | Sample size: 120 37 items (original version) 9 items (final version) | NR | RMSEA: 0.10 CFI: 0.97 | -1 |
| **Education Systems** | |  |  |  |  |  |
| CtI (52) | Overall: α=0.78 | 2 | Sample size: 351 2 items (and 12 items in other questionnaires) | NR | NR | 0 |
| SVS (36) | Overall scale: NR Importance of goals: α=0.66 Importance of effects: α=0.68 Importance of methods: α=0.64 | 1 | Sample size for validation: 306 23 items for initial version | NR | Factor structure child version:  CFI=0.962 RMSEA=0.026 | 3 |
| **Workplaces** |  |  |  |  |  |  |
| IOHORC (45) | Overall: α=0.77 α-values for subscales not reported | 2 | Sample size: 3703 8 items | NR | Four factor model differentiating between agents of change and all subscales; Full sample:  RMSEA: 0.036 CFI: 0.996 (95% CI: 0.027-0.045) Random sample (n=200) RMSEA: 0.00 CFI: 1.00 (95% CI: 0.00-0.076) | 3 |
| WHPCI (39) | Health promotion willingness scale: α=0.83 Health promotion management scale: α=0.91 | 3 | Sample size: 517 11 items | Explained variance (Model 3): Health promotion willingness: Explained variance: 59.1% Health promotion management: Explained variance: 75.1% | NR | 4 |
| **Different settings** | |  |  |  |  |  |
| GSE (55, 65, 66) | Overall (57): α=0.815 Overall (68): α=0.92 | 3 | Sample size (68):  10 items | Explained variance: 57.1% with 1 factor | NR | 4 |
| GLTSI (37, 40, 60, 61) | Range subscales: α=0.58-0.89 | 1 | Relation: 2.7-216.75 | Training-in-general domain: Explained variance: 50.1%  Training specific domain:  Explained variance: 62.17% | Training-in-general domain:  CFI=0.92 RMSEA=0.06 Training specific domain:  CFI=0.97 RMSEA=0.07 | 2 |
| PKSMHP (46) | Overall: α-values not reported  Perceived knowledge in project Management and planning skills: α=0.79 Perceived knowledge about tools, methods and services: α=0.82 Perceived knowledge of how to recognise and detect mental health problem in individuals: α=0.87 | 2 | Sample size: 106 37 items (original version) 9 items (final version) | NR for final version | RMSEA: 0.052 (90% CI: 0.000 - 0.099) CFI: 0.985 | 2 |
| SS-TC (44, 62) | Range overall: α=0.84-0.88 Range subscales: α=0.74-0.87 | 2 | Relation: 68.75 | NR | RMSEA: 0.06 (90% CI: 0.05-0.07) CFI: 0.96 SRMR: 0.04 | 2 |
