# Supplementary material for: German language questionnaires for assessing implementation constructs and outcomes of psychosocial and health-related interventions: a systematic review
Source: Implement Sci. 2018 Dec 12;13:150. doi: 10.1186/s13012-018-0837-3 (PMC6292038; doi:10.1186/s13012-018-0837-3)
Supplement: Supplementary file 3 — 1. Details psychometric criteria—reliability and structural validity. 2 Details psychometric criteria—construct validity. 3. Details psychometric criteria—criterion validity, test-retest reliability. 4. Details psychometric criteria—norms, usability. 5. Details psychometric criteria—face and content validity, responsiveness. (ZIP 158 kb) [file 13012_2018_837_MOESM3_ESM.zip › SIID_Additional File 3.3_PC_criterion validity test-retestR1.docx]

**Additional File 3.3:** Predictive and concurrent criterion validity, and test-retest reliability

| **Instrument** | **Criterion Validity - Predictive** | | **Criterion Validity - Concurrent** | | **Test-Retest Reliability** | |
| --- | --- | --- | --- | --- | --- | --- |
|  | correlation with future criterion | Rating | correlation with concurrent criterion | Rating | Adminis-tration period (2-14 days) | Cohen’s kappa; (κ) > 0.60  Pearson’s correlation; (r) > 0.70  Intra-Class Correlation; (ICC) > 0.70 |
| **Hospital and Health Care Setting** | |  |  |  |  |  |
| AMMHTA (53) | NR | 0 | NR | 0 | NR | NR |
| AGS (54) | NR | 0 | NR | 0 | NR | NR |
| APOI-HP (34) | NR | 0 | NR | 0 | NR | NR |
| APOI (38) | NR | 0 | NR | 0 | NR | NR |
| CSQ-I (33, 58) | NR | 0 | NR | 0 | NR | NR |
| CSQ-8 (59, 63, 64) | Based on study 2:  Psychological distress [SCL-90-R]: r=0.17 Interpersonal problems [IIP-D]: r=0.11 Life satisfaction [FLZ]: r=0.15 Physical functioning: [SF-36]: r=0.05 Psychological wellbeing [SF-36]: r=0.15 | 1 | Based on study 2: *Self-rating:* Satisfaction with success [HAQ]: r=0.64 Satisfaction with relationship [HAQ]: r=0.72 Psychological distress [SCL-90-R]: r=0.23 Interpersonal problems [IIP-D]: r=0.11 Life satisfaction [FLZ]: r=0.19 *Proxy-rating:* Level of physical impairment [BSS]: r=0.04 Level of psychological impairment [BSS]: r=0.10 Life satisfaction [BSS]: r=0.03 | 3 | NR | NR |
| CVF (55, 67) | NR | 0 | NR | 0 | NR | NR |
| DTSQ(C) (32, 56) | NR | 0 | NR | 0 | NR | NR |
| DTSQ(S) (32, 57) | NR | 0 | NR | 0 | NR | NR |
| EUUS (47) | NR | 0 | NR | 0 | NR | NR |
| EHRAS (41) | NR | 0 | Explained variance of the model: CFI=0.97; RMSEA=0.10; n=204 | 1 | NR | NR |
| EGIP (55, 67) | NR | 0 | NR | 0 | NR | NR |
| FraSiK (49) | NR | 0 | NR | 0 | NR | NR |
| GQ-TPB (30) | NR | 0 | Explained variance of the model (Attitude, subjective norm/perceived behaviour control and usage of arriba-lib, 8 weeks later): R^2^=0.12 | -1 | 14 days | Attitude: r=0.75 Subjective norm and Perceived behaviour control: r=0.41 |
| GUQ-DUR (50) | NR | 0 | NR | 0 | NR | NR |
| HSOPSC (43) | NR | 0 | Explained variance of the model on "Overall perceptions of safety": R^2^(adj)=0.539; n=562 Explained variance of the model on "Frequency of event report": R^2^(adj)=0.351; n=535 | 2 | NR | NR |
| KFPG (54) | NR | 0 | NR | 0 | NR | NR |
| OLS (55, 67) | NR | 0 | NR | 0 | NR | NR |
| PEACS (35) | NR | 0 | With subscale "Shared decision-making at indication" and the SDM-Q-9-scale: r=0.814; With subscale "Information at discharge and follow-up" and the CTM-3: r=0.511 | 3 | 3 weeks | Ranging from r=0.671 to r=0.855 |
| PUA-MSM (42) | NR | 0 | NR | 0 | NR | NR |
| SAMS-P and SAMS-S (51) | *SAMS-P*:  With QoL total parent rating improvement: r=0.39 With reduction of parent-rated ADHD symptoms total: r=0.43 With reduction of teacher-rated ADHD symptoms - Improvement in Clinical Global Impression (CGI): r=0.25 *SAMS-S:*  With QoL total child rating improvement: r=0.31 With QoL total adolescent rating improvement: r=0.30 With reduction of parent-rated ADHD symptoms total: r=0.28 With reduction of teacher-rated ADHD symptoms total: r=0.25 With improvement in Clinical Global Impression (CGI): r=0.22 | 1 | Assessment at visit 3: *SAMS-P:*  With parent ratings of ADHD symptoms total: r=-0.60 With Clinical Global Impression: r=-0.31  *SAMS-S:* With parent ratings of ADHD symptoms total: r=-0.38 With Clinical Global Impression: r=-0.25 | 2 | 3-10 weeks | *SAMS-P:* r=0.54 *SAMS-S:* r=0.59 |
| SOAPC (31) | NR | 0 | With Warr-Cook-Wall Scale (job satisfaction): r=0.499 | 2 | NR | NR |
| USE (48) | NR | 0 | NR | 0 | NR | NR |
| **Education Systems** | |  |  |  |  |  |
| CtI (52) | NR | 0 | NR | 0 | NR | NR |
| SVS (36) | NR | 0 | NR | 0 | NR | NR |
| **Workplaces** |  |  |  |  |  |  |
| IOHORC (45) | NR | 0 | Correlation of "readiness for change" score with Affective commitment: r=0.377 Self-efficacy: r=0.174 Job satisfaction: r=0.350 Self-related health: r=0.244 Exhaustion: r=-0.314 Work engagement: r=0.384 | 2 | NR | NR |
| WHPCI (39) | NR | 0 | NR | 0 | NR | NR |
| **Different settings** | |  |  |  |  |  |
| GSE (55, 65, 66) | NR | 0 | NR | 0 | NR | NR |
| GLTSI (37, 40, 60, 61) | NR | 0 | Explained variance of the model (including all subscales): Individual performance Improvement: R^2^(adj)=0.51 Number of taken steps: R^2^(adj)=0.15 Average implementation: R^2^(adj)=0.19 | -1 | NR | NR |
| PKSMHP (46) | NR | 0 | NR | 0 | NR | NR |
| SS-TC (44, 62) | NR | 0 | Frequency of technology usage:  Technology commitment: r=0.40  Technology acceptance: r=0.37 Technology competence: r=0.37 Technology control: r=0.17  Technology biography: r=0.31 Technology commitment: r=0.33  Technology acceptance: r=0.29 Technology competence: r=0.43  Technology control: r=0.02 | 2 | NR | NR |
