# Supplementary material for: German language questionnaires for assessing implementation constructs and outcomes of psychosocial and health-related interventions: a systematic review
Source: Implement Sci. 2018 Dec 12;13:150. doi: 10.1186/s13012-018-0837-3 (PMC6292038; doi:10.1186/s13012-018-0837-3)
Supplement: Supplementary file 3 — 1. Details psychometric criteria—reliability and structural validity. 2 Details psychometric criteria—construct validity. 3. Details psychometric criteria—criterion validity, test-retest reliability. 4. Details psychometric criteria—norms, usability. 5. Details psychometric criteria—face and content validity, responsiveness. (ZIP 158 kb) [file 13012_2018_837_MOESM3_ESM.zip › SIID_Additional File 3.2_PC_Construct ValidityR1.docx]

**Additional File 3.2:** Construct Validity (convergent validity, discriminant validity, known-group validity)

| **Instrument** | **Construct Validity - Convergent** | | **Construct Validity - Discriminant (divergent)** | | **Construct Validity - Known-Groups** | |
| --- | --- | --- | --- | --- | --- | --- |
|  | Constructs and Cohen's d | Rating | Constructs and  Cohen's d | Rating | Hypothesis of the difference, comparison goups, p-value | Rating |
| **Hospital and Health Care Setting** | |  |  |  |  |  |
| AMMHTA (53) | NR | 0 | NR | 0 | NR | 0 |
| AGS (54) | NR | 0 | NR | 0 | NR | 0 |
| APOI-HP (34) | NR | 0 | NR | 0 | NR | 0 |
| APOI (38) | NR | 0 | NR | 0 | NR | 0 |
| CSQ-I (33, 58) | NR | 0 | Study 1: with depressive symptoms after the intervention: r=-0.35; Cohen's d=-0.75 Study 2: with perceived stress after the intervention: r=-0.48, Cohen's d=-1.09 | 4 | NR | 0 |
| CSQ-8 (59, 63, 64) | With change in quality of life (r=0.52), Cohen's d=1.22 With current health status (r=0.43), Cohen's d=0.93 With current quality of life (r=0.40), Cohen's d=0.87 With assessment of vitality (r=0.39), Cohen's d=0.85 With external attribution of therapy success (r=0.21), Cohen's d=0.43 With internal attribution of therapy success (r=0.11), Cohen's d=0.22 Based on routine study: Overall quality index (r=0.84), Cohen's d=3.1 [quality of treatment (r=0.78), Cohen's d=2.5 Quality of housing (r=0.55), Cohen's d=1.32 Quality of catering (r=0.51), Cohen's d=1.19 Quality of leisure time and surrounding (r=0.58), Cohen's d=1.42 Changes in health (r=0.63), Cohen's d=1.62 Treatment (r=0.59), Cohen's d=1.46] Quality of life at the beginning (r=0.00), Cohen's d=0.00 Current quality of life (r=0.40), Cohen's d=0.87 Treatment options (r=0.64), Cohen's d=1.67 Treatment success (r=0.60), Cohen's d=1.5 | 4 | With quality of life at the beginning (r= -0.26), Cohen's d=-0.54 | 3 | NR | 0 |
| CVF (55, 67) | NR | 0 | NR | 0 | NR | 0 |
| DTSQ(C) (32, 56) | NR | 0 | NR | 0 | Using the DTSQc: 'At ceiling group' had greater increase in satisfaction than the 'Not at ceiling group' Using the DTSQDiff: 'Not at ceiling group' had greater increase in satisfaction than "At ceiling group" | 4 |
| DTSQ(S) (32, 57) | DTSQ(S) - 18-item version: With W-BQ total score: r=0.39 (p=0.008), Cohen's d=0.85 With energy: r=0.31 (p=0.038), Cohen's d=0.65 With positive well-being: r=0.44 (p=0.002), Cohen's d=0.98 With German measure of Health locus of control: With internality: r=0.49 (p<0.001), Cohen's d=1.12 With chance: r=-0.32 (p=0.002), Cohen's d=0.68 | 4 | DTSQ(S) - 18-item version: With mean blood glucose:  r= -0.31 (p=0.025), Cohen's d=-0.65 With depression: r=-0.29 (p=0.049), Cohen's d=-0.61 With unforeseeability r=-0.43 (p=0.001), Cohen's d=-0.93 | 4 | NR | 0 |
| EUUS (47) | NR | 0 | NR | 0 | NR | 0 |
| EHRAS (41) | NR | 0 | NR | 0 | NR | 0 |
| EGIP (55, 67) | NR | 0 | NR | 0 | NR | 0 |
| FraSiK (49) | NR | 0 | NR | 0 | NR | 0 |
| GQ-TPB (30) | NR | 0 | NR | 0 | NR | 0 |
| GUQ-DUR (50) | NR | 0 | NR | 0 | NR | 0 |
| HSOPSC (43) | NR | 0 | NR | 0 | NR | 0 |
| KFPG (54) | NR | 0 | NR | 0 | NR | 0 |
| OLS (55, 67) | NR | 0 | NR | 0 | NR | 0 |
| PEACS (35) | NR | 0 | NR | 0 | NR | 0 |
| PUA-MSM (42) | NR | 0 | NR | 0 | NR | 0 |
| SAMS-P and SAMS-S (51) | NR | 0 | NR | 0 | NR | 0 |
| SOAPC (31) | NR | 0 | NR | 0 | NR | 0 |
| USE (48) | Total scale with  "All in all, the brochure was useful to me": r=0.68, Cohen's d=1.85 "I will recommend the brochure": r=0.61, Cohen's d=1.54  “If needed, I'm going to read the brochure again": r=0.62, Cohen's d=1.58 | 4 | NR | 0 | Statistical significant better rating between the full version of the patient information material and the reduced material (d=-0.975; 95% CI: -1.354 - -0.597) | 3 |
| **Education Systems** | |  |  |  |  |  |
| CtI (52) | NR | 0 | NR | 0 | NR | 0 |
| SVS (36) | NR | 0 | NR | 0 | NR | 0 |
| **Workplaces** |  |  |  |  |  |  |
| IOHORC (45) | NR | 0 | NR | 0 | NR | 0 |
| WHPCI (39) | NR | 0 | NR | 0 | NR | 0 |

| **Instrument** | **Construct Validity - Convergent** | | **Construct Validity - Discriminant (divergent)** | | **Construct Validity - Known-Groups** | |
| --- | --- | --- | --- | --- | --- | --- |
|  | Constructs and Cohen's d | Rating | Constructs and  Cohen's d | Rating | Hypothesis of the difference, comparison goups, p-value | Rating |
| **Different settings** |  |  |  |  |  |  |
| GSE (55, 65, 66) | Total scale with Resilience (RS): 3 subscales ranging from r=0.62-0.68, Cohen's d=1.58-1.85 | 4 | Total scale with Health complaints (GBB-24): 5 subscales ranging from r=-0.24 - -0.33; Cohen's d=-0.49 - -0.70 Belief (SBI): r=0.13, Cohen's d=0.26 | 4 | NR | 0 |
| GLTSI (37, 40, 60, 61) | NR | 0 | NR | 0 | NR | 0 |
| PKSMHP (46) | NR | 0 | NR | 0 | NR | 0 |
| SS-TC (44, 62) | With sex (men vs. women): r=NR, Cohen's d=0.41-0.63 With education: r=0.07, Cohen's d=0.14 With usage of technical devices: r=0.31, Cohen's d=0.65 With openness to new experience: r=0.11 - 0.29, Cohen's d=0.22 - 0.61 With self-efficacy: r=0.37, Cohen's d=0.80 With life satisfaction: r=0.23, Cohen's d=0.47 With social functioning: r=0.17, Cohen's d=0.35 With attitude towards aging: r=0.31, Cohen's d=0.65 | 3 | With age: r=-0.18, Cohen's d=-0.37 With neuroticism: r=-0.21 - -0.23, Cohen's d=-0.43 - -0.47 With intelligence:  I-S-T 2000-R: r=0.00, Cohen's d=0.00 HAWIE-R-Number-Symbol-Test, r=-0.08, Cohen's d=-0.16 | 4 | NR | 0 |
