# Supplementary material for: German language questionnaires for assessing implementation constructs and outcomes of psychosocial and health-related interventions: a systematic review
Source: Implement Sci. 2018 Dec 12;13:150. doi: 10.1186/s13012-018-0837-3 (PMC6292038; doi:10.1186/s13012-018-0837-3)
Supplement: Supplementary file 3 — 1. Details psychometric criteria—reliability and structural validity. 2 Details psychometric criteria—construct validity. 3. Details psychometric criteria—criterion validity, test-retest reliability. 4. Details psychometric criteria—norms, usability. 5. Details psychometric criteria—face and content validity, responsiveness. (ZIP 158 kb) [file 13012_2018_837_MOESM3_ESM.zip › SIID_Additional File 3.4_PC_Norms usabilityR1.docx]

**Additional File 3.4:** Norms and usability

| **Instrument** | **Norms** | | **Usability** | | **Missing Items** |
| --- | --- | --- | --- | --- | --- |
|  | Sample Size, Norm Population | Rating | Number of Items | Rating |  |
| **Hospital and Health Care Setting** | |  |  |  |  |
| AMMHTA (53) | NR | 0 | 33 | 3 | NR |
| AGS (54) | General attitude toward guidelines: M=3.4; SD=0.527 Usefulness of guidelines: M=3.31; SD=0.546 Reliability of guidelines: M=3.23; SD=0.728 Lack of individual or team competence: M=2.99; SD=0.612 Lack of organisational competence: M=3.15; SD=0.614 Impracticality of guidelines: M=2.70; SD=0.677 Availability of guidelines: M=3.42; SD=0.540 Sample size (T3): n=107-110 | 2 | 14 | 3 | NR |
| APOI-HP (34) | Total Scale: M=45.14, SD=8.20, n=428 Scepticism and perception of risks: M=12.96, SD=3.00 Confidence in effectiveness: M=14.26, SD=2.98 Technologization threat: M=15.35, SD=2.5 Anonymity benefits: M=11.18, SD=2.6 | 3 | 16 | 3 | NR |
| APOI (38) | Values only reported on item-level | 0 | 16 | 3 | NR |
| CSQ-I (33, 58) | *Study 1:*  M=26.26; SD=5.34; n=174;  *Study 2:* M=26.05; SD=4.96; n=111 | 2 | 8 | 4 | NR |
| CSQ-8 (59, 63, 64) | M=27.00; SD=4.01; n=53177 | 4 | 8 | 4 | NR |
| CVF (55, 67) | NR | 0 | 27 | 3 | NR |
| DTSQ(C) (32, 56) | NR | 0 | 8 | 4 | NR |
| DTSQ(S) (32, 57) | NR | 0 | 8 | 4 | NR |
| EUUS (47) | Usefulness T0: M=20.6; SD=4.1 Usefulness T1: M=20.3; SD=5.1 Ease of use T0: M=21.7; SD=4.3 Ease of use T1: M=23.3; SD=3.9 Sample size: n=45 | 0 | 8 | 4 | NR |
| EHRAS (41) | Privacy concern: M=2.22; SD=0.66 Social influence: M=1.64; SD=0.85 Cost reduction: M=1.00; SD=0.91 Improvement: M=1.59; SD=0.81 Attitude: M=1.24; SD=0.94 Intention to use: M=1.27; SD=0.82 Sample size: n=204 | 2 | 28 | 3 | Max: 5%, others deleted |
| EGIP (55, 67) | NR | 0 | 27 | 3 | NR |
| FraSiK (49) | NR | 0 | 72 | 2 | Overall mean: 1.9% Max >10% |
| GQ-TPB (30) | NR | 0 | 41 | 3 | Item non-response:  Max: 5% |
| GUQ-DUR (50) | Use: M=2.61; SD=1.04 Attitude: M=2.12; SD=0.86 (corrected version: M=1.91; SD=0.98) Availability: M=2.94; SD=1.33 (corrected version: M=3.13; SD=1.36) Support: M=3.52; SD=1.34  Sample size: n=178 | 2 | Original version: 47  Extended version: 58 | 2 | NR |
| HSOPSC (43) | NR | 0 | 39 | 3 | NR |
| KFPG (54) | NR | 0 | 13 | 3 | NR |
| OLS (55, 67) | NR | 0 | 27 | 3 | NR |
| PEACS (35) | NR | 0 | 40 | 3 | Item non-response: 0.8%-9.3% |
| PUA-MSM (42) | NR | 0 | 27 | 3 | NR |
| SAMS-P and SAMS-S (51) | SAMS-P, visit 2: M=4.46; SD=1.03 SAMS-P, visit 3: M=4.69; SD=1.02 SAMS-S, visit 2: M=4.75; SD=0.86 SAMS-S, visit 3: M=4.87; SD=0.87  Sample size parents: n=589 Sample size patients: n=552 | 4 | 12 | 3 | NR |
| SOAPC (31) | Overall SOAPC score: M=3.81; SD=0.43 Communication: M=3.99; SD=0.63 Decision making: M=4.13; SD=0.55 Stress/chaos: M=3.57; SD=0.73 History of change: M=3.58; SD=0.65 Sample size: n=297 | 2 | 21 | 3 | Item non-response:  0.34%-3.03% |
| USE (48) | NR | 0 | 9 | 4 | NR |
| **Education Systems** | |  |  |  |  |
| CtI (52) | Commitment to innovation: M=3.44; SD=0.52 sample size: n=351 | 3 | 2 | 4 | NR |
| SVS (36) | NR | 0 | 17 | 3 | NR |
| **Workplaces** |  |  |  |  |  |
| IOHORC (45) | NR | 0 | 8 | 4 | 4.2% - 4.5% |
| WHPCI (39) | Health promotion willingness scale: M=5.66; SD=2.24 Health promotion management scale: M=2.33; SD=2.68 Sample size: n=517 | 4 | 9 | 4 | item non-response: 0.2%-3.1% |
| **Different settings** | |  |  |  |  |
| GSE (55, 65, 66) | Self-efficacy scale: M=29.4; SD=5.4 Age- and gender-adjusted norms available Sample size: n=2019 | 4 | 10 | 3 | NR |

| **Instrument** | **Norms** | | **Usability** | | **Missing Items** |
| --- | --- | --- | --- | --- | --- |
|  | Sample Size, Norm Population | Rating | Number of Items | Rating |  |
| GLTSI (37, 40, 60, 61) | *5 subscales for the training general domain:*  Performance self-efficacy: M=3.91; SD=0.58 Transfer effort - performance expectations: M=3.67; SD=0.66 Performance - outcome expectations: M=3.28; SD=0.88 Openness to change: M=3.59; SD=0.6 Performance feedback: M=2.91; SD=0.83 *11 subscales for training specific domain:* Learner readiness: M=2.94; SD=0.93 Motivation to transfer: M=3.51; SD=0.89 Transfer design: M=3.82; SD=0.70 Content validity: M=3.38; SD=0.81 Personal outcomes positive: M=2.28; SD=1.04 Personal outcomes negative: M=1.74; SD=0.78 Peer support: M=3.1; SD=0.82 Supervisor/manager support: M=2.71; SD=0.98 Supervisor/manager sanctions: M=1.97; SD=0.80 Personal capacity: M=3.40; SD=0.91 Opportunity to use: M=3.19; SD=0.83 Sample size: n=408 | 3 | 67 items | 2 | NR |
| PKSMHP (46) | Perceived knowledge in project management and Planning skills: M=7.19; SD=2.39 Perceived knowledge about tools, methods and services: M=9.07; SD=2.18 Perceived knowledge of how to recognise and detect mental health problem in individuals: M=8.46; SD=2.39 Sample size: n=106 | 2 | 9 | 4 | 0%-13.2% |
| SS-TC (44, 62) | Overall: M=3.73; SD=0.62 Technology acceptance: M=3.27; SD=0.94 Technology competence: M=4.16; SD=0.80 Technology control: M=3.75; SD=0.74 Sample size: n=825 | 4 | 12 | 3 | NR |
