# Supplementary material for: German language questionnaires for assessing implementation constructs and outcomes of psychosocial and health-related interventions: a systematic review
Source: Implement Sci. 2018 Dec 12;13:150. doi: 10.1186/s13012-018-0837-3 (PMC6292038; doi:10.1186/s13012-018-0837-3)
Supplement: Supplementary file 3 — 1. Details psychometric criteria—reliability and structural validity. 2 Details psychometric criteria—construct validity. 3. Details psychometric criteria—criterion validity, test-retest reliability. 4. Details psychometric criteria—norms, usability. 5. Details psychometric criteria—face and content validity, responsiveness. (ZIP 158 kb) [file 13012_2018_837_MOESM3_ESM.zip › SIID_Additional File 3.5_PC_Face Validity ResponsivR1.docx]

**Additional File 3.5:** Face and content validity, and responsiveness

| **Instrument** | **Face and Content Validity** | | | **Responsiveness** |  |
| --- | --- | --- | --- | --- | --- |
|  | Theory or Framework | Items Adapted from Previous Instruments | Face/Content Validation | Change in Pre-Post-Measurement | Rating |
| **Hospital and Health Care Setting** | |  |  |  |  |
| AMMHTA (53) | Technology Acceptance Model (Davis, 1985) | 9 different Instruments: Venkatesh, 2003; Pedersen, 2001; Nysveen, 2005; Wixom, 2005; Bagozzi, 2002; Park, 2009; Jarupathirun, 2007; Gu, 2009; Chau, 2002; Gheong, 2005; | NR | NR | 0 |
| AGS (54) | NR | English version of "The Attitudes towards Guidelines Scales (AGS)" (Elovaino, 1999) | NR | NR | 0 |
| APOI-HP (34) | NR | APOI-HP Items adopted from APOI | NR | NR | 0 |
| APOI (38) | Literature review; Ajzen Framework of attitudes | Credibility/ Expectancy Questionnaire (CEQ; Devilly and Borkovec, 2000) Psychotherapy Expectations, Concerns, and Hopes Inventory (PECHI; Moritz et al., 2012b) | Authors approved face validity | NR | 0 |
| CSQ-I (33, 58) | NR | CSQ-8 English Version (Attkinsson, 1982) | Focus group of the research group and feedback from experts in the field | NR | 0 |
| CSQ-8 (59, 63,64) | Client satisfaction seen as one relevant outcome (Donabedian 1966) | CSQ-8 English Version (Attkinsson, 1982) | NR | NR | 0 |
| CVF (55, 67) | NR | Goh and Richards, 1997 | NR | NR | 0 |
| DTSQ(C) (32, 56) | NR | DTSQ(S) 8-item version | NR | Direct measurement of change | 0 |
| DTSQ(S) (32, 57) | NR | Items adapted from English version | NR | NR | 0 |
| EUUS (47) | Technology Acceptance Model (Davis, 1989) | Examining the technology acceptance model using physician acceptance of telemedicine technology (Hu et al., 1999) | NR | NR | 0 |
| EHRAS (41) | Technology Acceptance Model, Unified Theory of Acceptance and Use of Technology; Theory of Reasoned Action | No | Feedback of 15 Austrian physicians participating in a pre-test was incorporated into the final instrument | NR | 0 |
| EGIP (55, 67) | NR | Caldwell, 2004 | NR | NR | 0 |
| FraSiK (49) | Organizational Culture and Leadership (Schein, 1987) | Safety Attitudes Questionnaire, Ambulatory Version (SAQ-A) (Harkness and Blom, 2006) | Expert interviews in the development phase, literature-based; cognitive interviews with draft version | NR | 0 |
| GQ-TPB (30) | Theory of planned behaviour (Ajzen, 1991) | No | Qualitative interviews to elicit GPs beliefs about facilitators and barriers of adhering to complex interventions; pre-test of the questionnaire | NR | 0 |
| GUQ-DUR (50) | Literature analysis was conducted for the original version | Utilization Questionnaire - Dissemination and Use of Research (Champion and Leach, 1989) | NR | NR | 0 |
| HSOPSC (43) | NR | Translation of Hospital Survey on Patients Safety Culture (Sorra and Nieva, 2004) | Translation and back-translation by different translators; inconsistencies were solved by discussions; pre-test with clinical and non-clinical staff | NR | 0 |
| KFPG (54) | NR | No | Delphi groups consisting of four experts | NR | 0 |
| OLS (55, 67) | NR | Shortell et al., 2004 | NR | NR | 0 |
| PEACS (35) | COSMIN initiative (quality criteria for health-related patient-reported outcomes) | No | Stepwise development process | NR | 0 |
| PUA-MSM (42) | Technology Acceptance Model | Chismar et al., 2002; Bhattacherjee et al., 2007; Moore et al., 1991 | NR | NR | 0 |
| SAMS-P and SAMS-S (51) | NR | No | NR | NR | 0 |
| SOAPC (31) | Medical Home or Chronic Care Model | Items translated from the English version | Translation and backward translation, consensus meeting with the authors of the original SOAPC | NR | 0 |
| USE (48) | Tripartite model of reading for evaluating and enhancing patient information material (Garner et al., 2012) | No | Preliminary item pool was rated by 18 independent researchers | NR | 0 |
| **Education Systems** | |  |  |  |  |
| CtI (52) | NR | Quality Assurance in School and Curriculum (QuaSSU, Ditton, 2011) | NR | NR | 0 |
| SVS (36) | Framework of social validity ratings for prevention programmes | No | Feedback from children and teachers was considered | NR | 0 |
| **Workplaces** |  |  |  |  |  |
| IOHORC (45) | Theory of planned behaviour (Ajzen, 1991) | No | No | NR | 0 |
| WHPCI (39) | Bandura's social learning theory | No | Stepwise development process, focus groups | NR | 0 |
| **Different settings** | |  |  |  |  |
| GSE (55, 65, 66) | Theory of Self-Efficacy | No | NR | NR | 0 |
| GLTSI (37, 40, 60, 61) | Training-Transfer-Model extended by influencing factors defined by Noe and Schmitt (1986) | Items translated from the English version | Forward and back translations as well as subjective evaluations | NR | 0 |
| PKSMHP (46) | Framework of steps that need to be taken when designing and implementing a programme (Barry and Jenkins, 2007) | No | Expert rating of items | NR | 0 |
| SS-TC (44, 62) | Technology Acceptance Model | Scales to measure “technical assessment” and “technical experience” (Mollenkopf and Kaspar, 2004) | Pre-test of instrument | NR | 0 |
