# Supplementary material for: German language questionnaires for assessing implementation constructs and outcomes of psychosocial and health-related interventions: a systematic review
Source: Implement Sci. 2018 Dec 12;13:150. doi: 10.1186/s13012-018-0837-3 (PMC6292038; doi:10.1186/s13012-018-0837-3)
Supplement: Supplementary file 4 — Overview of instrument’s psychometric properties used in settings. (DOCX 27 kb) [file 13012_2018_837_MOESM4_ESM.docx]

**Additional File 4: Overview of instrument’s psychometric properties used in settings**

| **Psychometric Properties** | **Hospital & Health Care** | | | | **Education System** | | | | **Workplaces** | | | | **Multiple Settings** | | | | **Overall** | | | |
| --- | --- | --- | --- | --- | --- | --- | --- | --- | --- | --- | --- | --- | --- | --- | --- | --- | --- | --- | --- | --- |
|  | n | % | M | SD | n | % | M | SD | n | % | M | SD | n | % | M | SD | n | % | M | SD |
| Internal consistency | 22 | 96 | 1.7 | 1.5 | 2 | 100 | 1.5 | 0.5 | 2 | 100 | 2.5 | 0.5 | 4 | 100 | 2.0 | 0.7 | 30 | 97 | 1.8 | 1.4 |
| Convergent Validity | 3 | 13 | 0.5 | 1.3 | 0 | 0 | 0.0 | 0.0 | 0 | 0 | 0.0 | 0.0 | 2 | 50 | 1.8 | 1.8 | 5 | 16 | 0.6 | 1.4 |
| Discriminant Validity | 3 | 13 | 0.5 | 1.2 | 0 | 0 | 0.0 | 0.0 | 0 | 0 | 0.0 | 0.0 | 2 | 50 | 2.0 | 2.0 | 5 | 16 | 0.6 | 1.4 |
| Known-Groups Validity | 2 | 8.7 | 0.3 | 1.0 | 0 | 0 | 0.0 | 0.0 | 0 | 0 | 0.0 | 0.0 | 0 | 0 | 0.0 | 0.0 | 2 | 6.5 | 0.2 | 0.9 |
| Predictive Validity | 2 | 8.7 | 0.1 | 0.3 | 0 | 0 | 0.0 | 0.0 | 0 | 0 | 0.0 | 0.0 | 0 | 0 | 0.0 | 0.0 | 2 | 6.5 | 0.1 | 0.2 |
| Concurrent Validity | 7 | 30 | 0.5 | 1.1 | 0 | 0 | 0.0 | 0.0 | 1 | 50 | 1.0 | 1.0 | 2 | 50 | 0.3 | 1.1 | 10 | 32 | 0.5 | 1 |
| Structural Validity | 14 | 61 | 1.1 | 1.8 | 1 | 50 | 1.5 | 1.5 | 2 | 100 | 3.5 | 0.5 | 4 | 100 | 2.5 | 0.9 | 21 | 68 | 1.5 | 1.8 |
| Responsiveness | 0 | 0 | 0.0 | 0.0 | 0 | 0 | 0.0 | 0.0 | 0 | 0 | 0.0 | 0.0 | 0 | 0 | 0.0 | 0.0 | 0 | 0 | 0 | 0 |
| Norms | 8 | 35 | 0.9 | 1.3 | 1 | 50 | 1.5 | 1.5 | 1 | 50 | 2.0 | 2.0 | 4 | 100 | 3.3 | 0.8 | 14 | 45 | 1.3 | 1.6 |
| Usability | 23 | 100 | 3.2 | 0.6 | 2 | 100 | 3.5 | 0.5 | 2 | 100 | 4.0 | 0.0 | 4 | 100 | 3.0 | 0.7 | 31 | 100 | 3.2 | 0.6 |
| Test-retest Reliability | 3 | 13 | 0.1 | 0.3 | 0 | 0 | 0.0 | 0.0 | 0 | 0 | 0.0 | 0.0 | 0 | 0 | 0.0 | 0.0 | 3 | 9.7 | 0.1 | 0.3 |
| Face & Content Validity | 22 | 96 | 1.0 | 0.2 | 2 | 100 | 1.0 | 0.0 | 2 | 100 | 1.0 | 0.0 | 3 | 75 | 0.8 | 0.4 | 29 | 94 | 0.9 | 0.2 |

**Abbreviations:** M, mean overall ratings; n, number and percentage of instruments with at least a rating of 1; %, percentage of instruments with at least a rating of 1; SD, standard deviation overall ratings
