# Supplementary material for: German language questionnaires for assessing implementation constructs and outcomes of psychosocial and health-related interventions: a systematic review
Source: Implement Sci. 2018 Dec 12;13:150. doi: 10.1186/s13012-018-0837-3 (PMC6292038; doi:10.1186/s13012-018-0837-3)
Supplement: Supplementary file 5 — Mapping CFIR and IOF constructs. (DOCX 66 kb) [file 13012_2018_837_MOESM5_ESM.docx]

**Additional Material 5: Mapping of scales and subscales against the CFIR and IOF constructs**

|  | **CFIR - Intervention Characteristics** | | | | | | | | **CFIR - Outer Setting** | | | | **CFIR - Inner Setting** | | | | | | | | | | | | | | **CFIR - Character-istics of individuals** | | | | **CFIR - Process** | | | | | | | | **IOF** | | | | | | | | **# Subscales** | | |
| --- | --- | --- | --- | --- | --- | --- | --- | --- | --- | --- | --- | --- | --- | --- | --- | --- | --- | --- | --- | --- | --- | --- | --- | --- | --- | --- | --- | --- | --- | --- | --- | --- | --- | --- | --- | --- | --- | --- | --- | --- | --- | --- | --- | --- | --- | --- | --- | --- | --- |
|  |  |  |  |  |  |  |  |  |  |  |  |  |  |  |  |  |  |  |  |  |  |  |  |  |  |  |  |  |  |  |  |  |  |  |  |  |  |  |  |  |  |  |  |  |  |  |  |  |  |
| Instrument | Intervention Source | Evidence Strength & Quality | Relative Advantage | Adaptability | Trialability | Complexity | Design Quality & Packaging | Cost | Patient Needs & Resources | Cosmopolitanism | Peer Pressure | External Policy & Incentives | **Structural Characteristics** | **Networks & Communications** | **Culture** | **Implementation Climate (IC)** | IC: Tension for Change | IC: Compatibility | IC: Relative Priority | IC: organizational incentives & Rewards | IC: Goals and Feedback | IC: Learning Climate | **Readiness for Implementation (RI)** | RI: Leadership Engagement | RI: Available Resources | R1: Access to Knowledge/Information | Knowledge & Beliefs | Self-efficacy | Individual Stage of Change | Identification with Organisation | Planning | Engaging (EN) | EN: Opinion Leaders | EN: Internal Implementation Leaders | EN: Champions | EN: External Change Agents | Executing | Reflecting & Evaluating | Acceptability | Adoption | Appropriateness | Cost | Feasibility | Fidelity | Penetration | Sustainability | # Subscales CFIR | # Subscales IOF | # Subscales Overall |
| **Hospital and Health Care Setting** | | | | | | | | |  |  |  |  |  |  |  |  |  |  |  |  |  |  |  |  |  |  |  |  |  |  |  |  |  |  |  |  |  |  |  |  |  |  |  |  |  |  | **26** | **21** | **47** |
| AMMHTA |  |  |  |  |  |  |  |  |  |  |  |  |  |  | † |  |  |  |  |  |  |  |  |  |  |  |  | † |  |  |  |  |  |  |  |  |  |  | † |  | † |  | † |  |  |  | 2 | 3 | 5 |
| AGS |  | † |  |  |  |  |  |  |  |  |  |  |  |  |  |  |  |  |  |  |  |  |  |  | † |  |  |  |  |  |  |  |  |  |  |  |  |  | † |  | † |  | † |  |  |  | 2 | 3 | 5 |
| APOI-HP |  |  |  |  |  |  |  |  |  |  |  |  |  |  |  |  |  |  |  |  |  |  |  |  |  |  |  |  |  |  |  |  |  |  |  |  |  |  | † |  |  |  |  |  |  |  | 0 | 1 | 1 |
| APOI |  |  |  |  |  |  |  |  |  |  |  |  |  |  |  |  |  |  |  |  |  |  |  |  |  |  |  |  |  |  |  |  |  |  |  |  |  |  | † |  |  |  |  |  |  |  | 0 | 1 | 1 |
| CSQ-I |  |  |  |  |  |  |  |  |  |  |  |  |  |  |  |  |  |  |  |  |  |  |  |  |  |  |  |  |  |  |  |  |  |  |  |  |  |  | † |  |  |  |  |  |  |  | 0 | 1 | 1 |
| CSQ-8 |  |  |  |  |  |  |  |  |  |  |  |  |  |  |  |  |  |  |  |  |  |  |  |  |  |  |  |  |  |  |  |  |  |  |  |  |  |  | † |  |  |  |  |  |  |  | 0 | 1 | 1 |
| CVF |  |  |  |  |  |  |  |  |  |  |  |  |  |  | † |  |  |  |  |  |  |  |  |  |  |  |  |  |  |  |  |  |  |  |  |  |  |  |  |  |  |  |  |  |  |  | 1 | 0 | 1 |
| DTSQ(C) |  |  |  |  |  |  |  |  |  |  |  |  |  |  |  |  |  |  |  |  |  |  |  |  |  |  |  |  |  |  |  |  |  |  |  |  |  |  | † |  |  |  |  |  |  |  | 0 | 1 | 1 |
| DTSQ(S) |  |  |  |  |  |  |  |  |  |  |  |  |  |  |  |  |  |  |  |  |  |  |  |  |  |  |  |  |  |  |  |  |  |  |  |  |  |  | † |  |  |  |  |  |  |  | 0 | 1 | 1 |
| EUUS |  |  |  |  |  |  |  |  |  |  |  |  |  |  |  |  |  |  |  |  |  |  |  |  |  |  |  |  |  |  |  |  |  |  |  |  |  |  | † |  |  |  | † |  |  |  | 0 | 2 | 2 |
| EHRAS |  |  |  |  |  |  |  |  |  |  | † |  |  |  |  |  |  |  |  |  |  |  |  |  |  |  | † |  |  |  |  |  |  |  |  |  |  |  |  |  |  |  |  |  |  |  | 2 | 0 | 2 |
| EGIP |  |  |  |  |  |  |  |  |  |  |  |  |  |  |  |  |  |  |  |  |  |  |  |  |  |  |  |  |  |  |  |  |  |  |  |  |  | † |  |  |  |  |  |  |  |  | 1 | 0 | 1 |
| FraSiK |  |  |  |  |  |  |  |  |  |  |  |  |  | † |  |  |  |  |  |  |  | † |  | † |  |  |  |  |  |  |  |  |  |  |  |  |  |  |  |  |  |  |  |  |  |  | 3 | 0 | 3 |
| GQ-TPB |  |  |  |  |  |  |  |  |  |  |  |  |  |  |  |  |  |  |  |  |  |  |  |  |  |  | † |  |  |  |  |  |  |  |  |  |  |  | † |  |  |  |  |  |  |  | 1 | 1 | 2 |
| GUQ-DUR |  |  |  |  |  |  |  |  |  |  |  |  |  | † |  |  |  |  |  |  |  |  |  |  | † |  |  |  |  |  |  |  |  |  |  |  |  |  | † |  |  |  |  |  |  |  | 2 | 1 | 3 |
| HSOPSC |  |  |  |  |  |  |  |  |  |  |  |  |  | † | † |  |  |  |  |  |  | † |  | † |  |  |  |  |  |  |  |  |  |  |  |  |  |  |  |  |  |  |  |  |  |  | 4 | 0 | 4 |
| KFP |  |  |  |  |  |  |  |  |  |  |  |  |  |  |  |  |  |  |  |  |  |  |  |  |  |  | † |  |  |  |  |  |  |  |  |  |  |  |  |  |  |  |  |  |  |  | 1 | 0 | 1 |
| OLS |  |  |  |  |  |  |  |  |  |  |  |  |  | † |  |  |  |  |  | † |  |  |  | † |  |  |  |  |  |  |  |  |  |  |  |  |  |  |  |  |  |  |  |  |  |  | 3 | 0 | 3 |
| PEACS |  |  |  |  |  |  |  |  |  |  |  |  |  |  |  |  |  |  |  |  |  |  |  |  |  |  |  |  |  |  |  |  |  |  |  |  |  |  | † |  |  |  |  |  |  |  | 0 | 1 | 1 |
| PUA-MSM |  | † |  |  |  |  |  |  |  |  |  |  |  |  |  |  | † | † |  |  |  |  |  |  |  |  |  |  |  |  |  |  |  |  |  |  |  |  | † |  |  |  | † |  |  |  | 3 | 2 | 5 |
| SAMS-P and SAMS-S |  |  |  |  |  |  |  |  |  |  |  |  |  |  |  |  |  |  |  |  |  |  |  |  |  |  |  |  |  |  |  |  |  |  |  |  |  |  | † |  |  |  |  |  |  |  | 0 | 1 | 1 |
| SOAPC |  |  |  |  |  |  |  |  |  |  |  |  |  | † |  |  |  |  |  |  |  |  |  |  |  |  |  |  |  |  |  |  |  |  |  |  |  |  |  |  |  |  |  |  |  |  | 1 | 0 | 1 |
| USE |  |  |  |  |  |  |  |  |  |  |  |  |  |  |  |  |  |  |  |  |  |  |  |  |  |  |  |  |  |  |  |  |  |  |  |  |  |  | † |  |  |  |  |  |  |  | 0 | 1 | 1 |
| **Education Systems** | | | | |  |  |  |  |  |  |  |  |  |  |  |  |  |  |  |  |  |  |  |  |  |  |  |  |  |  |  |  |  |  |  |  |  |  |  |  |  |  |  |  |  |  | 1 | 1 | 2 |
| CtI |  |  |  |  |  |  |  |  |  |  |  |  |  |  |  |  |  |  |  |  |  |  |  | † |  |  |  |  |  |  |  |  |  |  |  |  |  |  |  |  |  |  |  |  |  |  | 1 | 0 | 1 |
| SVS |  |  |  |  |  |  |  |  |  |  |  |  |  |  |  |  |  |  |  |  |  |  |  |  |  |  |  |  |  |  |  |  |  |  |  |  |  |  |  |  | † |  |  |  |  |  | 0 | 1 | 1 |
| **Workplace Settings** | | | | |  |  |  |  |  |  |  |  |  |  |  |  |  |  |  |  |  |  |  |  |  |  |  |  |  |  |  |  |  |  |  |  |  |  |  |  |  |  |  |  |  |  | 4 | 0 | 4 |
| IOHORC |  |  |  |  |  |  |  |  |  |  |  |  |  |  |  |  | † |  |  |  |  |  |  |  |  |  | † |  |  |  |  |  |  |  |  |  |  |  |  |  |  |  |  |  |  |  | 2 | 0 | 2 |
| WHPCI |  |  |  |  |  |  |  |  |  |  |  |  |  |  |  |  |  |  | † |  |  |  |  |  |  |  |  |  |  |  |  |  |  |  |  |  | † |  |  |  |  |  |  |  |  |  | 2 | 0 | 2 |
| **Multiple Settings** | | | |  |  |  |  |  |  |  |  |  |  |  |  |  |  |  |  |  |  |  |  |  |  |  |  |  |  |  |  |  |  |  |  |  |  |  |  |  |  |  |  |  |  |  | 10 | 2 | 12 |
| GSE |  |  |  |  |  |  |  |  |  |  |  |  |  |  |  |  |  |  |  |  |  |  |  |  |  |  |  | † |  |  |  |  |  |  |  |  |  |  |  |  |  |  |  |  |  |  | 1 | 0 | 1 |
| GLTSI |  |  |  |  |  |  |  |  |  |  |  |  |  | † |  |  |  |  |  | † |  | † |  | † |  |  | † | † | † |  |  |  |  |  |  |  |  |  |  |  |  | † |  |  |  |  | 7 | 1 | 8 |
| PKSMHP |  |  |  |  |  |  |  |  |  |  |  |  |  |  |  |  |  |  |  |  |  |  |  |  |  |  |  |  |  |  |  |  |  |  |  |  |  |  |  |  |  |  |  |  |  |  | 0 | 0 | 0 |
| SS-TC |  |  |  |  |  |  |  |  |  |  |  |  |  |  |  |  |  |  |  |  |  |  |  |  |  |  | † | † |  |  |  |  |  |  |  |  |  |  | † |  |  |  |  |  |  |  | 2 | 1 | 3 |
| **Summe** | **0** | **2** | **0** | **0** | **0** | **0** | **0** | **0** | **0** | **0** | **1** | **0** | **0** | **6** | **3** | **0** | **2** | **1** | **1** | **2** | **0** | **3** | **0** | **5** | **2** | **0** | **6** | **4** | **1** | **0** | **0** | **0** | **0** | **0** | **0** | **0** | **1** | **1** | **16** | **0** | **3** | **1** | **4** | **0** | **0** | **0** | **41** | **24** | **65** |

**Abbreviations:** CFIR, Consolidated Framework for Implementation Research; EN, Engaging; IC, Implementation Climate; IOF, Implementation Outcome Framework; RI, Readiness for Implementation;
